# CO_2_ Reduction: From the Electrochemical to Photochemical Approach

**DOI:** 10.1002/advs.201700194

**Published:** 2017-09-12

**Authors:** Jinghua Wu, Yang Huang, Wen Ye, Yanguang Li

**Affiliations:** ^1^ Institute of Functional Nano and Soft Materials (FUNSOM) Jiangsu Key Laboratory for Carbon‐Based Functional Materials and Devices Soochow University Suzhou 215123 China

**Keywords:** CO_2_ reduction, electrocatalysis, nanotechnology, photocatalysis

## Abstract

Increasing CO_2_ concentration in the atmosphere is believed to have a profound impact on the global climate. To reverse the impact would necessitate not only curbing the reliance on fossil fuels but also developing effective strategies capture and utilize CO_2_ from the atmosphere. Among several available strategies, CO_2_ reduction via the electrochemical or photochemical approach is particularly attractive since the required energy input can be potentially supplied from renewable sources such as solar energy. In this Review, an overview on these two different but inherently connected approaches is provided and recent progress on the development, engineering, and understanding of CO_2_ reduction electrocatalysts and photocatalysts is summarized. First, the basic principles that govern electrocatalytic or photocatalytic CO_2_ reduction and their important performance metrics are discussed. Then, a detailed discussion on different CO_2_ reduction electrocatalysts and photocatalysts as well as their generally designing strategies is provided. At the end of this Review, perspectives on the opportunities and possible directions for future development of this field are presented.

## 1 Introduction

**Figure 1:**
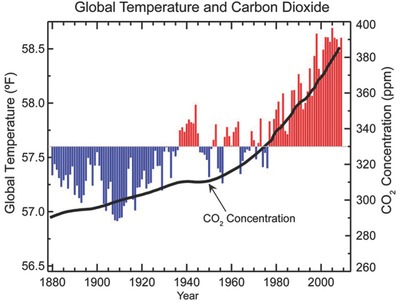
Atmospheric CO_2_ concentration and corresponding global average temperature since the late 19th century. Red bars indicate temperatures above and blue bars indicate temperatures below the 1901–2000 average temperature. Adopted from the website of National Ocean and Atmospheric Administration (NOAA).^3^ Copyright 2017, NOAA.

Energy shortage and environmental pollution are two major global challenges facing the human society. Current world energy consumption is highly dependent upon fossil fuels. Concerns are growing that the increasing human activities would not only accelerate the consumption of fossil fuels but also result in escalated greenhouse gas emission and breaks the carbon balance in the natural world.^1^, ^2^ Since late 19th century, CO_2_ concentration in the atmosphere has increased from 280 to 400 ppm (**Figure** **1**).^3^ This has resulted in the continuous rise of the global average temperature. How to effectively reduce the atmospheric CO_2_ level and further utilize it has become an important research topic worldwide. Strategies are now being actively sought to mitigate CO_2_ emission via improving the combustion efficiency of fossil fuels or exploring clean and renewable energy sources (e.g., wind, tide, and solar energy).^4^, ^5^ Alternatively, great efforts are also being actively undertaken to develop carbon capture and storage (CCS) techniques that fix atmospheric CO_2_ and store it underground in a supercritical state.^6^ However, the CCS technique itself is energy intensive and nonrenewable.

**Figure 2:**
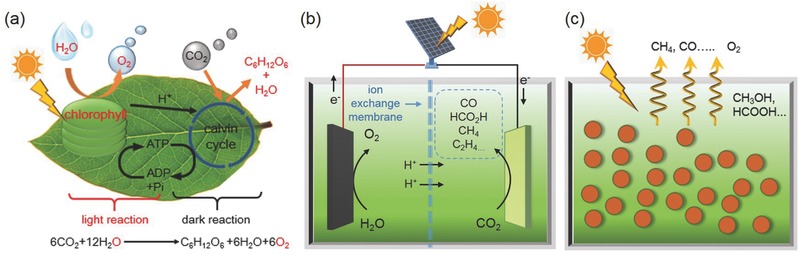
Analogy among a) natural photosynthesis, b) electrochemical synthesis on electrocatalysts powered by a photovoltaic cell, and c) photochemical synthesis on powdery photocatalysts.

In nature, the photosynthesis of green plants plays an indispensable role in maintaining the carbon/oxygen cycle which is vital for the maintenance of life on earth. It is consisted of two sequential steps known as the light and dark reactions (**Figure** **2**a).^7^ In the light reaction, chlorophyll adsorbs sunlight, converts it to the chemical energy stored in adenosine triphosphate (ATP), and meanwhile oxidizes water to O_2_. In the dark reaction, CO_2_ is fixed and reduced stepwise to form carbohydrates using energy stored in ATP. The natural photosynthesis essentially provides the energy needed for most lives on this planet, and is the basis for the survival of mankind.

For more than three decades, researchers have been ambitiously attempting to mimic what Mother Nature does and pursuing artificial photosynthesis that aims at the spontaneous transformation of atmospheric CO_2_ and water to chemical fuels using sunlight as the sole energy input.^8^, ^9^ Although still at very low efficiency currently, artificial photosynthesis is believed to have the great potential to make a substantial contribution to our future energy supply. It is now generally approached from two directions. As schematically illustrated in Figure 2b, the first route uses photovoltaic (PV) cells to generate a sufficient photovoltage which is then supplied to the cathode for the CO_2_ reduction and the anode for the water oxidation. Proper electrocatalysts are employed on the two electrodes so as to expedite the reaction rate and improve the reaction selectivity. The advantage of this route is the flexibility in the design of PV and electrocatalyst pairs. Components can be individually optimized and then combined together to enable the best overall performance. The second route is the direct photocatalytic approach where light‐absorbing semiconductor particles (photocatalysts) decorated with suitable electrocatalysts (commonly referred as cocatalysts in photocatalysis) are dispersed in aqueous solution and achieve light harvesting, charge separation, and interfacial charge transfer to drive corresponding reactions all within particles (Figure 2c). The merit of the second route is its wireless configuration that renders the device design much more straightforward and compact. At this moment, it is too early to judge which route would eventually dominate in the future. Their future success strongly relies on the development of high‐performance CO_2_ reduction electrocatalysts or photocatalysts.

Since CO_2_ is a thermodynamically stable molecule, its multistep reduction via the electrochemical or photochemical approach is significantly more challenging than the splitting of water, and confronted with many fundamental technical hurdles.^10^ The history of electrocatalytic CO_2_ reduction can be traced back to 19th century. In 1870, Royer first reported the reduction of CO_2_ to formic acid on Zn electrodes.^11^ Between 1970s and 1980s, a series of seminal works published by Japanese scientists marked the advent of a new phase in electrocatalytic CO_2_ reduction research. Ito and Murata examined the electrocatalytic performances of several metals such as In, Cd, Sn, Zn, and Pb for reducing CO_2_ to formic acid.^12^ Hori et al. discovered that polycrystalline Cu electrodes in aqueous media could generate short‐chain hydrocarbons with a promising activity.^13^ Studies on photocatalytic CO_2_ reduction commenced in 1970s. In 1978, Halmannn first observed that CO_2_ was reduced to CH_3_OH and CO on a p‐type GaP electrode under light illumination.^14^ In the year later, Inoue et al. reported that formic acid, formaldehyde, and methyl alcohol were produced from the photocatalytic reduction of CO_2_ in the aqueous suspensions of semiconductors such as TiO_2_, ZnO, CdS, GaP, and SiC.^15^ Following these pioneering works on electrocatalytic or photocatalytic CO_2_ reduction, increasing attention has been attracted to this field, and many exciting progresses have been made in recent years.

In this paper, we present an overview of the recent progress on electrocatalytic or photocatalytic CO_2_ reduction. Several previous high‐quality review articles on similar topics are available.^16^, ^17^, ^18^, ^19^, ^20^, ^21^, ^22^, ^23^, ^24^, ^25^, ^26^ Given the recent heightened research activities and increasingly deepened understanding of these two processes, we believe that an up‐to‐date account on their status and existing challenges is necessary so as to provide readers with a current snapshot of this rapidly evolving area. Even though the two approaches involve dissimilar experimental techniques, their nature is essentially identical—that is how to activate the chemically inert CO_2_ molecule and promote its conversion under external energy stimuli. In addition, the surface charge transfer step in photocatalysis is in fact an electrochemical process and can be enhanced via the incorporation of proper cocatalysts. These are the reasons why we think electrocatalytic and photocatalytic CO_2_ reduction are inherently connected and decide to discuss them together here. This review starts with a brief description about the basic principles and important performance merits of electrocatalytic and photocatalytic CO_2_ reduction. It is then followed by detailed discussions on different catalysts for electrocatalytic and photocatalytic CO_2_ reduction and their several designing strategies. At the end, we present our brief perspectives on the possible future development of this field.

## 2 Fundamentals of Electrocatalytic and Photocatalytic CO_2_ Reduction

CO_2_ is one of the most stable molecules due to the strong C=O double bond with bonding energy of 750 kJ mol^−1^—considerably larger than that of C—C (336 kJ mol^−1^), C—O (327 kJ mol^−1^), or C—H bond (411 kJ mol^−1^). CO_2_ reduction via either the electrocatalytic or the photocatalytic approach is a thermodynamically uphill reaction and demands significant energy input to break the C=O bond. To make it even more complicated, CO_2_ reduction may proceed via several different reaction pathways with the transfer of 2, 4, 6, 8, 12 or even more electrons and yielding diverse reduction products including carbon monoxide (CO), formic acid (HCOOH), methane (CH_4_), ethylene (C_2_H_4_), and many others depending on the nature of the electrocatalysts or photocatalysts as well as the actual experimental conditions.^27^, ^28^ As a result, electrocatalytic or photocatalytic CO_2_ reduction is generally suffered from very limited efficiency and poor selectivity.

### 2.1 Fundamentals of Electrocatalytic CO_2_ Reduction

**Figure 3:**
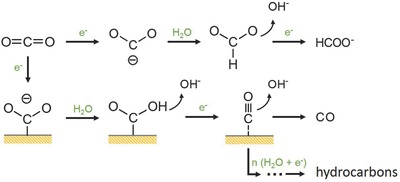
Possible reaction pathways for electrocatalytic CO_2_RR on metal electrodes in aqueous solutions. Adapted with permission.^45^ Copyright 1994, Elsevier.

**Table** **1** summarizes the equilibrium potentials (vs the standard hydrogen electrode, SHE) of CO_2_ reduction to different products in pH 7.0 aqueous solution.^29^ Even though some reactions (i.e., reduction to CH_4_, methanol or C_2_H_4_) are thermodynamically more favorable than the two‐electron hydrogen evolution reaction (HER), the kinetics of the CO_2_ reduction is substantially more sluggish. This is because after its chemical absorption on the working electrode, the first electron transfer to form CO_2_^•−^ anion radical does not initiate until at—1.90 V in order to reorganize the originally linear molecule into a bent anion radical (**Figure** **3**).^30^, ^31^ The formation of this intermediate state imposes a significant overpotential to the reaction and is frequently identified as the rate determining step.^32^, ^33^ Once CO_2_^•−^ is formed, it may be subsequently reduced via the protonation of its oxygen atom, resulting in the formation of ^•^COOH. This intermediate is then reduced to CO and released from the electrode surface. Alternatively, CO_2_^•−^ may also be reduced via the protonation of its carbon atom to form HCOO^•^ instead of ^•^COOH at high overpotentials, which is further reduced to formate (HCOO^−^). As a result, most CO_2_ reduction electrocatalysts yield CO or formate as the primary reduction products. Only on very few electrocatalysts (e.g., Cu), CO can be further reduced to hydrocarbons. The reaction mechanism of these electrocatalysts is not clearly understood, but it is believed to proceed stepwise via the H addition, the scission of C—O bond, and the coupling of C—C bond.^34^, ^35^, ^36^

**Table 1:** Standard electrochemical potentials for CO_2_ reduction

Reduction potentials of CO_2_	*E*° [V] vs SHE at pH 7
CO_2_ + e^−^ → CO_2_^−^	−1.9
CO_2_ + 2H^+^ + 2e^−^ → HCOOH	−0.61
CO_2_ + 2H^+^ + 2e^−^ → CO + H_2_O	−0.52
2CO_2_ + 12H^+^ + 12e^−^ → C_2_H_4_ + 4H_2_O	−0.34
CO_2_ + 4H^+^ + 4e^−^ → HCHO + H_2_O	−0.51
CO_2_ + 6H^+^ + 6e^−^ → CH_3_OH + H_2_O	−0.38
CO_2_ + 8H^+^ + 8e^−^ → CH_4_ + 2H_2_O	−0.24
2H^+^ + 2e^–^ → H_2_	−0.42

There are several important performance metrics in the evaluation of CO_2_ reduction electrocatalysts as listed in the following:

*Onset potential*: onset potential refers to the working potential where the electrocatalytic current starts to take off from the background. It is not a well‐defined parameter since sometimes it is difficult to determine the exact current onset point especially when the capacitive contribution is significant. To avoid the confusion, onset potential now is frequently defined as the potential where the electrocatalytic current of a certain product reaches a given level (e.g., 0.1 mA cm^−2^). Such a level also varies from study to study. Care therefore needs to be taken when comparing the onset potential across the literature.
*Tafel slope*: Tafel slope (*b*) can be experimentally derived by plotting the overpotential (η) with respect to the logarithm of the current density (*log j*) and fitting the linear region of the curve with the Tafel equation (η = *b log j + a*). It indicates the overpotential increment necessary to raise the current density of a certain product by tenfold. A smaller Tafel slope corresponds to a steep rise of the current density with the increasing overpotential and is a highly desirable characteristic of electrocatalysts. For multielectron transfer process such CO_2_ reduction reaction, the Tafel slope may also provide valuable insights into the rate determining step and possible reaction pathway. For example, when the formation of CO_2_^•–^ anion radical is rate determining, the ideal Tafel slope should be $b=\;\frac{2.3RT}{\alpha \;F}=118\;mV$ per decade.^37^
*Turnover frequency (TOF)*: TOF is defined as the rate of electrochemical conversions per electrocatalytic site at certain overpotential. It reflects the intrinsic activity of an electrocatalyst and allows the comparison among different materials regardless of their actual geometric parameters or areal loading. Unfortunately, except for a few special cases where material surface activity sites can be clearly quantified (e.g., Pt and Pd),^38^ it is highly challenging to calculate the TOF value of most electrocatalysts due to the structural ambiguity of the active sites and the difficulty in precisely counting them. Many studies often assume all the added catalysts effectively participate in the reaction. Thus derived TOF values are underestimated (sometime by orders of magnitude), but they may still provide some insights into the intrinsic activity.
*Faradaic efficiency*: Faradaic efficiency of a certain product is defined as the ratio of charges transferred to this product relative to the total charges passed through the circuit. It reflects the selectivity of electrocatalysts. Since CO_2_ reduction kinetics is slow, it is usually accompanied by the considerable cogeneration of H_2_ from HER. High Faradaic efficiency (>80%) toward the desirable products is one of the many requirements for good CO_2_ reduction electrocatalysts.
*Stability*: Besides its activity and selectivity, any electrocatalyst should have sufficient long‐term stability in order to be considered for practical applications. It is usually evaluated via the cyclic voltammetry (CV) cycling, galvanostatic or potentiostatic polarizations. Evaluating the stability of electrocatalysts and understanding their possible degradation mechanism is a critical step toward the continuous optimization of electrocatalysts.

### 2.2 Fundamentals of Photocatalytic CO_2_ Reduction

**Figure 4:**
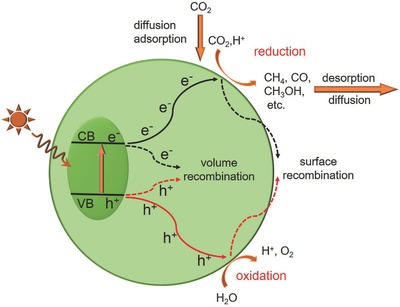
Schematic showing the five fundamental steps in photocatalytic CO_2_ reduction. Adopted with permission.^20^ Copyright 2014, Springer.

A typical process of photocatalytic CO_2_ reduction on a semiconductor photocatalyst is schematically illustrated in **Figure** **4**.^20^ It consists of five sequential steps—light absorption, charge separation, CO_2_ adsorption, surface redox reaction, and product desorption. The first step is the absorption of photons to generate electron and hole pairs. Illumination of a photocatalyst with the incident light excites electrons from the valance band (VB) to the conduction band (CB), leaving an equal number of holes in VB. In order for these photogenerated electrons or holes to be energetically favorable to reduce CO_2_ or oxidize water, photocatalysts should possess suitable band structure. Their CB edge must be more negative than the redox potential of CO_2_ reduction (summarized in Table 1), and the VB edge should be more positive than the redox potential of water oxidation (0.817 V vs SHE in pH 7.0 aqueous solution). The band gap has to be sufficiently large since we need to additionally take into consideration of the large overpotentials associated with these two electrochemical reactions. On the other hand, the band gap of photocatalysts cannot be too large as this would limit their effective utilization of the solar spectrum. For example, one of the most well studied semiconductors—TiO_2_ has a band gap of 3.2 eV. It only absorbs photons of light in the ultraviolet domain (<400 nm), which accounts for less than 5% of the entire solar spectrum.^39^ Given these two criteria, the ideal band gap is estimated to be 1.8–2.0 eV. However, most photocatalyst materials at present have band gaps off the ideal range. Strategies such as doping and solid solution are being actively sought to carefully engineer photocatalyst band structures as will be discussed later.

The second step is the spatial separation of photogenerated electrons and holes. This process is in direct competition with the charge recombination. Whether the charge separation is dominant over the recombination or vice versa depends on the relative time scale between the lifetime of photogenerated carriers and the recombination rate and is determined by a complex interplay among material crystallinity, dimension, surface properties, and many other structural factors. Pronounced recombination would result in the significant loss of free charge carriers and the release of harvested energy as heat. To enhance the overall photocatalytic efficiency therefore requires improving the separation efficiency of photogenerated carriers and suppressing their possible recombination. It may be achieved via the proper modification of material structures, such as selective surface treatments.

The third step is the CO_2_ adsorption. It is the prerequisite for the electron transfer from the photocatalyst to the CO_2_ molecule. In general, photocatalysts with high surface areas can provide more active sites for the CO_2_ adsorption. Another possible route to improve the CO_2_ adsorption is the alkali modification of the photocatalyst surface, as has been demonstrated for TiO_2_.^40^, ^41^ Due to the Lewis acidity of CO_2_ molecules, the reaction between CO_2_ and the alkaline photocatalyst surface would lead to the formation of intermediates such as bidentate carbonate, favoring the activation and subsequent reduction of CO_2_ molecules.

The forth step is the surface redox reaction. After migrating to the surface, photogenerated electrons and holes can separately drive different half reactions: electrons for reducing CO_2_ to CO, CH_4_, HCOOH, CH_3_OH or other hydrocarbons, and holes for oxidizing water to molecular O_2_. This step is a purely electrochemical process. The introduction of cocatalysts for CO_2_ reduction or water oxidation would dramatically enhance the interfacial charge transfer rate, and hence the overall solar‐to‐fuel conversion efficiency (SFE). Optimal electrocatalysts identified from electrochemical studies are also good candidates as cocatalysts, given that a suitable interface is established between the semiconductor photocatalyst and cocatalyst to enable the rapid charge transfer across it. This step also clearly reflects the interesting connection between electrocatalysis and photocatalysis. After the photocatalytic reaction is complete, the last step is the product desorption. If the product cannot be timely released from the catalyst surface, the reaction then is terminated and the catalyst becomes “poisoned.”

For photocatalytic CO_2_ reduction, the reaction can be carried out in liquid or gas phase medium. Most current studies refer the gas phase medium, where the suspended powdery photocatalyst directly reacts with surrounding CO_2_ and water vapor under light irradiation. The activity of photocatalysts are usually quantified using the production rate of a certain product (in terms of µmol h^−1^, or µmol h^−1^ g^−1^ when normalized with respect to the mass of photocatalyst) under given conditions including temperature (usually room temperature), pressure (usually 1 atm), and light intensity (usually 1 sun or 100 mW cm^−2^). TOF values of photocatalysts are similarly calculated based on the specific surface area of catalyst powders. Selectivity of photocatalysts is assessed by comparing the relative production rates of different gaseous or liquid products (including H_2_). In addition, photocatalysis has the following two important performance metrics that are frequently cited in literature:

*Apparent quantum efficiency (AQE) or external quantum efficiency (EQE)*: AQE or EQE is defined as the number ratio of electrons transferred toward a certain product relative to incident photons at a given wavelength. They can be expressed as the product of the efficiencies of light absorption, charge separation, and surface redox reaction. Photocatalysts therefore have to be efficient at all the three steps in order to have great AQE or EQE values.
*SFE*: SFE is defined as the ratio of converted chemical energy relative to the incident solar energy. It can also be understood as the integral of AQE or EQE over the entire solar spectrum. By comparison, AQE or EQE reflects the energy conversion efficiency of photocatalysts at a particular wavelength, while SFE reflects the overall energy conversion efficiency of photocatalysts. The ideal limiting SFE at a single absorber particle is η = 14.4% based on a light absorber with a 2.0 eV band gap.^42^ It is suggested that a >10% SFE is required for photocatalysis to be an economically viable resource.^43^, ^44^

One critical issue that needs special attention for the CO_2_ reduction research is the possible carbon contamination. Studies suggest that organic substances including solvents, reactants, and surfactants used for the catalyst preparation may leave carbonaceous residues in the final product, and, during electrocatalysis or photocatalysis (particularly the latter), may decompose to small molecules such as CO and CH_4_, causing the overestimation of catalytic activities.^16^ It is therefore necessary to confirm that the measured products are indeed from the CO_2_ reduction rather than the decomposition of carbonaceous residues. Isotopic ^13^CO_2_ labeling is an effective technique to verify the origin of reduction products and has been widely employed in many studies. Additionally, the possible carbon contamination may also be ruled out by carrying out control experiments in an inert gas environment (N_2_ or Ar) under otherwise identical conditions. Compared to isotopic ^13^CO_2_ labeling, control experiments in N_2_ or Ar are generally more cost and time effective.

## 3 Electrocatalytic Materials for CO_2_ Reduction

In this part, we aim to review different electrocatalysts that have been developed for CO_2_ reduction reaction (CO_2_RR) in recent years. They can be generally categorized into metals, metal chalcogenides and carbonaceous materials (**Table** **2**). In what follows, we will review the current development status of these materials.

**Table 2:** Summary of CO_2_ reduction electrocatalysts from recent literature

Electrocatalyst	Electrolyte	Selectivity and activity	Stability	Reference
Cu NCs with 44 nm edge length	0.1 m KHCO_3_	*J*_tot_ = ≈5.7 mA cm^−2^, F.E. CO_2_RR 80%, ethylene 41%, methane 20% @ −1.1 V vs RHE	–	53
Cu mesopore electrode (width/depth)	0.1 m KHCO_3_	*J*_tot_ = 14.3 mA cm^−2^, F.E. C_2_H_4_ 38% (30 nm/40 cm) C_2_H_6_ 46%(30 nm/70 nm) @ −1.7 V vs NHE; onset potential −0.96 V vs NHE	–	226
3D porous hollow fiber Cu electrode	0.3 m KHCO_3_	*J*_tot_ = ≈10 mA cm^−2^, F.E. CO 75% @ −0.4 V vs RHE	24 h @ −0.4 V vs RHE	227
Cu NPs 13.1 nm	0.1 m KHCO_3_	*J*_tot_ = 20 mA cm^−2^, H_2_ 0.078, CO 0.016, CH_4_ 0.0018, C_2_H_4_ 0.0006 (Vol. % cm^−2^) @ −1.1 V vs RHE	–	54
Cu NPs	0.1 m NaHCO_3_	*J*_tot_ = ≈9 mA cm^−2^, F.E. CH_4_ 80%, H_2_ 13% @ −1.25 V vs RHE	1 h @ −1.25 V vs RHE	55
OD Cu films	0.5 m NaHCO_3_	*J*_tot_ = 2.7 mA cm^−2^, F.E. CO ≈40%, HCO_2_H 33% @ −0.5 V vs RHE	7 h @ −0.5 V vs RHE	48
Plasma‐activated Cu	0.1 m KHCO_3_	F.E. C_2_H_4_ 60% @ −0.9 V vs RHE; onset E: −0.5 V vs RHE	–	59
OD Au NPs	0.5 m NaHCO_3_	*J*_tot_ = 6 mA cm^−2^, F.E. CO 98% @ −0.4 V vs RHE	8 h @ −0.4 V vs RHE	65
Au_25_ cluster	0.1 m KHCO_3_	*J*_tot_ = ≈14.3 mA cm^−2^, F.E. CO 99.6% @ −0.89 V vs RHE	–	228
Au NPs 8 nm	0.5 m KHCO_3_	F.E. CO 90% @ −0.67 V vs RHE	–	63
Au NWs	0.5 m KHCO_3_	*J*_tot_ = 1.84 A g^−1^, F.E. CO 94% @ −0.35 V vs RHE	12 h @ −0.35 V vs RHE	67
Au/carbon nanotubes (CNT)	0.5 m NaHCO_3_	*J*_tot_ = 15 A g^−1^, F.E. CO 94% @ −0.5 V vs RHE	12 h @ −0.5 V vs RHE	66
Nanoporous Ag	0.5 m KHCO_3_	*J*_tot_ = 18 mA cm^−2^, F.E. CO ≈92% @ −0.6 V vs RHE	2 h @ −0.6 V vs RHE	74
Mesostructured Ag	0.1 m KHCO_3_	F.E. CO > 80% @ −0.7 V vs RHE	–	75
Oxide‐derived Ag	0.1 m KHCO_3_	*J*_tot_ = 1.15 mA cm^−2^, F.E. CO ≈89% @ −0.8 V vs RHE	–	73
Anodized polycrystalline Ag	0.1 m aqueous KHCO_3_	*J*_tot_ = 1.15 mA cm^−2^, F.E. CO ≈89% @ −0.8 V vs RHE	–	106
Graphene confined Sn quantum sheets	0.1 m NaHCO_3_	*J*_tot_ = 21.1 mA cm^−2^, F.E. HCOOH 89% @ −1.8 V vs SHE	18 h @ −1.8 V vs RHE	79
Sn/SnO*_x_* on Ti foil	0.5 m NaHCO_3_	F.E. CO ≈40.6% HCOOH ≈56.8% @ −0.7 V vs RHE	–	65
≈5 nm Sn NPs/graphene	0.1 m NaHCO_3_	*J*_tot_ = 10.2 mA cm^−2^ *_,_* F.E. HCOOH 93.6% @ −1.8 V vs SHE	18 h @ −1.8 V vs SHE	105
Au_3_Cu	0.1 m KHCO_3_	*J*_tot_ = 3 mA cm^−2^, F.E. CO 64.7% HCOOH 3.11% @ −0.73 V vs RHE	–	92
Cu‐In	0.1 m KHCO_3_	*J*_tot_ = 0.53 mA cm^−2^, F.E. CO 90% @ −0.5 V vs RHE	7 h @ −0.6 V vs RHE	93
Pd Icosahedra/C	0.1 m KHCO_3_	F.E. CO 91.1% @ −0.8 V vs RHE	10 h @ −0.9 V vs RHE	229
Mo‐Bi bimetallic chalcogenide	1‐butyl‐3‐methylimidazolium tetrafluoroborate (BMIM‐BF_4_) in MeCN	*J*_tot_ = 12.1 mA cm^−2^, F.E. MeOH 71.2% @ −0.7 V vs SHE	–	104
Vertically aligned Mo_0.95_Nb_0.05_S_2_	50 vol % 1‐ethyl‐3‐methylimidazolium tetrafluoroborate (EMIM‐BF_4_) and water	*J*_tot_ = 237 mA cm^−2^, F.E. CO 82% @ −0.8 V vs RHE	–	103
Bulk MoS_2_	96 mol% water and 4 mol% EMIM‐BF_4_	*J*_tot_ = ≈65 mA cm^−2^, F.E. CO ≈98% @ −0.764 V vs RHE	–	101
WSe_2_	50 mol% water and 50 mol% EMIM‐BF_4_	*J*_tot_ = 330 mA cm^−2^, F.E. CO ≈85% @ −0.764 V vs RHE	–	102
Boron‐doped diamond (BDD)	MeOH containing tetrabutylammonium perchlorate (TBAP)	*J*_tot_ = ≈0.1 mA cm^−2^, F.E. HCHO ≈74% @ −1.7 V vs Ag/Ag^+^	20 h @ −1.7 V vs Ag/Ag^+^	108
Polyethylenimine‐Nitrogen doped carbon nanotubes (PEI‐NCNT)	0.1 m KHCO_3_	*J*_tot_ = −9.5 mA cm^−2^, F.E. HCOOH 87% @ −1.8 V vs SCE	24 h @ −1.8 V vs SCE	105
polyacrylonitrile (PAN)‐based CNFs	EMIM‐BF_4_	*J*_tot_ = 3.86 mA cm^−2^, F.E. CO 98% @ −0.573 V vs SHE	9 h @ −0.573 V vs SHE	107
N‐doped nanodiamond/Si rod array	0.5 m NaHCO_3_	*J*_tot_ = −0.69–0.89 mA cm^−2^, F.E. CH_3_COO^−^ 91.2–91.8% @ −0.8–−1.0 V vs RHE	–	109
N‐doped graphene quantum dots (QDs)	1 m KOH	*J*_tot_ = 100 mA cm^−2^, total F.E. for CO_2_RR products 90% C_2_H_4_ 46% C_2_H_5_OH 21% CO 23% @ −0.86 V vs RHE	–	110

### 3.1 Metals

Elemental metals are among the earliest investigated CO_2_RR electrocatalysts. In a series of seminal works published between 1980s and 1990s, Hori et al. first reported that CO, CH_4_, formate, and other hydrocarbons were detected from the electrocatalytic CO_2_RR on various metal electrodes in aqueous KHCO_3_ electrolyte solution.^13^, ^27^, ^45^ Based on the reduction products, these metals are divided to three groups. The first group includes Sn, Pb, Bi, In, etc. They hardly adsorb the CO_2_^•−^ intermediate. Desorbed CO_2_^•−^ tends to be protonated at the carbon atom and ultimately transforms to formate or formic acid as the major reduction product. The second group includes Au, Ag, Zn, Pd, Ga, etc. They can bind the CO_2_^•−^ intermediate, catalyze the cleavage of C—O bond in CO_2_, and allow resultant CO to easily desorb from the electrode as the major reduction product. The third group includes Pt, Ti, Ni, Fe, etc. They have low HER overpotentials and strong CO adsorption properties, giving rise to H_2_ as the major production. In addition to the three groups, Cu is the only elemental metal capable of producing C_1_–C_3_ hydrocarbons at significant rates. It is suggested that the adsorption of CO on Cu is suitable for its further reduction to hydrocarbons or alcohols at high overpotentials through COH or CHO intermediates.^36^

Theoretical simulation has been proven a powerful tool to understand the electrocatalytic CO_2_RR activity and selectivity on different metals. Nørskov and co‐workers used density functional theory (DFT) calculations to describe trends in catalytic activity for CO_2_ reduction to CO as a function of the adsorption energies of the two reaction intermediates—COOH and CO.^46^ They revealed that on Au and Ag, the reaction rate was limited by CO_2_ activation, and resultant CO desorbed easily from their surfaces; whereas on Pd, Ni, Pt, and Rh, CO_2_ activation and conversion to adsorbed CO was facile, and the reaction rate was mainly limited by the desorption of CO due to its strong affinity. Cu had intermediate bonding strength with both CO and COOH in comparison with others. Unfortunately, all the metals were suggested to be well off the ideal activity since the CO and COOH adsorption energies were essentially linearly correlated, making it impractical to adjust one parameter without affecting the other. Studt and co‐workers compared the CO_2_ reduction to formic acid and its competing reactions on 27 different metal surfaces and found that Pb (211) surface was one of the most promising monometallic surfaces for the reduction of CO_2_ to formic acid with a virtually zero overpotential and very high selectivity, while Cd, Tl, and Sn surfaces were selective toward formic acid production but required relatively high overpotentials (0.2–0.4 V).^47^

#### 3.1.1 Copper

**Figure 5:**
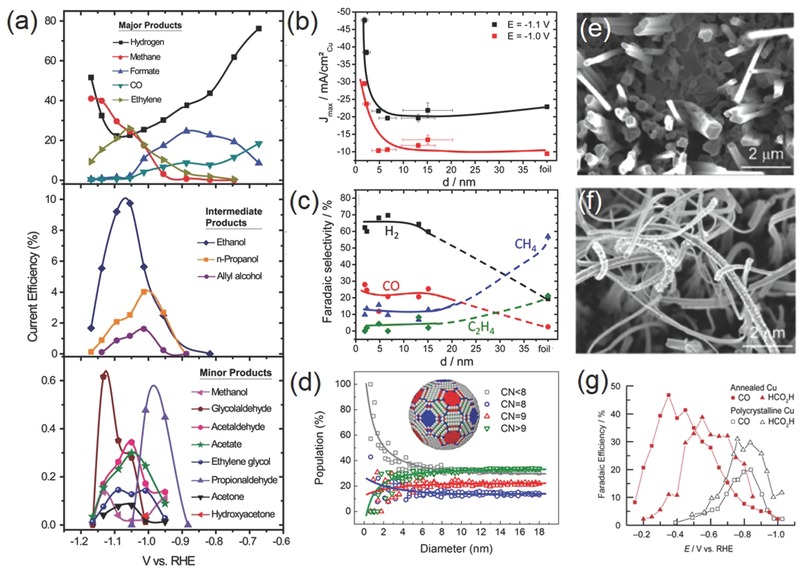
a) Faradaic efficiency as a function of potential for major (top), intermediate range (middle), and minor (bottom) products on a metallic Cu surface. Reproduced with permission.^36^ Copyright 2012, The Royal Society of Chemistry. Particle size dependence of b) current density and c) Faradaic efficiency for different CO_2_RR products on Cu NPs; d) population of surface atoms with certain coordination number (CN) as a function of particle diameter. Reproduced with permission.^54^ Copyright 2014, American Chemical Society. Scanning electron microscope (SEM) images of e) an annealed Cu electrode and f) the same electrode after CO_2_RR; g) Faradaic efficiency for CO and HCOOH as a function of potential on polycrystalline Cu and annealed Cu. Reproduced with permission.^48^ Copyright 2012, American Chemical Society.

As introduced above, Cu is the only known elemental metal that can reduce CO_2_ beyond CO or formic acid. In addition to the two‐electron reduction products, methane, ethane, ethylene, ethyne, methanol, and ethanol are all measured as the possible reduction products. Recent work by Jaramillo and co‐workers reported the identification of 16 different reduction products on metallic Cu surface (**Figure** **5**a).^36^ Aside from common products, some aldehydes, ketones, carboxylic acids, esters, and hydrocarbons such as paraffins and olefins containing up to six carbon atoms could also be generated as minor products on Cu electrocatalysts. It was proposed that these C_≥2_ products might be formed via enol‐like surface intermediates. Nevertheless, deep reduction of CO_2_ is kinetically challenging: these higher value chemicals generally cannot be produced at significant rates until at very negative potentials (←0.8 V vs reversible hydrogen electrode or RHE) and their Faradaic efficiency is usually <30%.^34^, ^36^, ^48^

To promote the electrocatalytic performance of Cu, different strategies have been exploited. The morphology of Cu catalysts has a profound influence on the catalytic activity and product selectivity. On single crystal Cu electrodes, the selectivity toward hydrocarbons (mainly methane and ethylene) is strongly dependent on the electrode surface. Hori et al. first showed that ethylene formation was favored on Cu (100) surface, whereas methane was the main hydrocarbon product on Cu (111) terraces.^49^ Similar results were also observed by Koper and co‐workers.^50^, ^51^ It is generally accepted that CO is a key intermediate in the formation of both methane and ethylene. Even though the exact reaction mechanism remains elusive, experimental evidence was shown by Koper and co‐workers that ethylene was formed on Cu (100) surface presumably via electron‐mediated dimerization reaction of two adsorbed CO molecules.^52^ Dimerization of CO on Cu (111) surface was unfavorable compared to the protonation of CO to CHO, which subsequently led to the formation of methane. However, a different insight was provided by Novskov and co‐workers suggesting that the formation of ethylene instead proceeded through the chemical dimerization of CHO intermediate based on DFT calculations.^35^ In spite of the debatable reaction pathway, this unique morphological dependence of hydrocarbon selectivity can be capitalized in the design and engineering of practical Cu‐based CO_2_RR electrocatalysts. For example, Nilsson and co‐workers reported a simple in‐situ synthesis of nanocube‐covered Cu surface having predominant (100) exposure for efficient and selective ethylene production.^53^ Its ethylene selectivity was measured to increase by more than two orders of magnitude compared to polycrystalline Cu with nearly complete suppression of the methane signal.

Particle size is also an important structural parameter for Cu‐based CO_2_RR electrocatalysts. Strasser and co‐workers discovered that Cu nanoparticles (NPs) exhibited dramatically enhanced total current density and higher selectivity toward CO and H_2_ as their particle size was decreased, particularly for those under 5 nm, while hydrocarbon selectivity was increasingly suppressed (Figure 5b–d).^54^ This experimental observation was rationalized by DFT calculations, which showed that smaller Cu NPs could provide more undercoordinated atoms as strong binding sites to key intermediates such as H and COOH, thus accelerating HER and the reduction of CO_2_ to CO while decreasing further recombination reaction to hydrocarbons. However, conflicting results were also disclosed by Alivisatos and co‐workers showing that Cu NPs (≈7 nm, grew to ≈25 nm during electrochemical experiments) exhibited an enhanced methanation current density four times greater than that of Cu foil, and an average Faradaic efficiency of 80% during extended electrolysis.^55^ The marked difference in reaction selectivity might be caused by the different synthetic approaches and measurement conditions employed.

Furthermore, surface roughening of Cu electrode materials is an effective route to promote their CO_2_RR performance. This is not only owing to the enlarged surface area but also due to the generation of a significant number of active surface sites such as edges, steps, and defects, which are suggested to have lower energy barriers for the formation of key CO_2_ reduction intermediates (e.g., CO and CHO).^56^ Indeed, evidence from thermal desorption spectroscopy demonstrated that CO bind considerably strongly onto Cu step edges, kinks, or defects than terrace sites.^57^ Surface roughening of Cu can be achieved in several different ways such as thermal annealing or electropolishing. Li and Kanan roughened Cu foils by first annealing them in air and then electrochemically reducing formed microthick Cu_2_O films to Cu nanoparticles (Figure 5e–g).^48^ Comparing to polycrystalline Cu, they observed that roughened Cu electrodes were capable of efficient CO_2_ reduction to CO and HCOOH at much lower overpotentials with greater current density and stability. In a follow‐up study, the same research group revealed that the roughening process generated a high density of grain boundaries which could support surface active sites normally unstable on individual nanoparticles.^58^ Cuenya and co‐workers employed facile and tunable plasma treatments to roughen Cu surfaces, and found that the optimal sample demonstrated a lower overpotential (−0.5 V vs RHE) and record selectivity (60% at −0.9 V) toward ethylene.^59^ Besides larger surface area and the increasing number of low‐coordinated sites, the authors suggested that the stable oxide layer formed during plasma treatment played a key role for enhancing the reaction activity and ethylene selectivity.

Aside from aforementioned electrode structural parameters, electrolyte cations or anions that were once thought to be idle may directly or indirectly impact the electrocatalytic process. It was not until very recently that their roles in CO_2_RR received careful investigations. Based on their experimental observation, Bell and co‐workers proposed that the p*K*_a_ for cation hydrolysis decreased from Li^+^ to Cs^+^ in their bicarbonate electrolyte, and larger cations such as K^+^, Rb^+^, and Cs^+^ had p*K*_a_ values sufficiently low that they could act as buffering agents and lower the local pH near the cathode, leading to increased ethylene selectivity and lowered Faradaic efficiency for H_2_ on electropolished Cu foils.^60^ The CO_2_RR performance of Cu can also be tuned with the addition of halide anions to the electrolyte. Strasser and co‐workers demonstrated that adding Cl^−^ and Br^−^ led to an increased CO selectivity compared with the halide‐free electrolyte.^61^ By contrast, in the presence of I^–^ the CO selectivity declined and methane formation was enhanced up to six times. It was suggested that adsorbed I^–^ anions on the cathode could favor the protonation of CO intermediate to CHO—a key step toward methane formation.

#### 3.1.2 Gold

Among various bulk metals, Au generally exhibits the highest activity and selectivity for reducing CO_2_ to CO,^45^, ^62^ and has attracted much attention over the last five years. Current research efforts are mainly centered on the development of nanostructured Au materials with further improved CO_2_RR performance and the understanding of its possible reaction mechanism at the atomic level.

**Figure 6:**
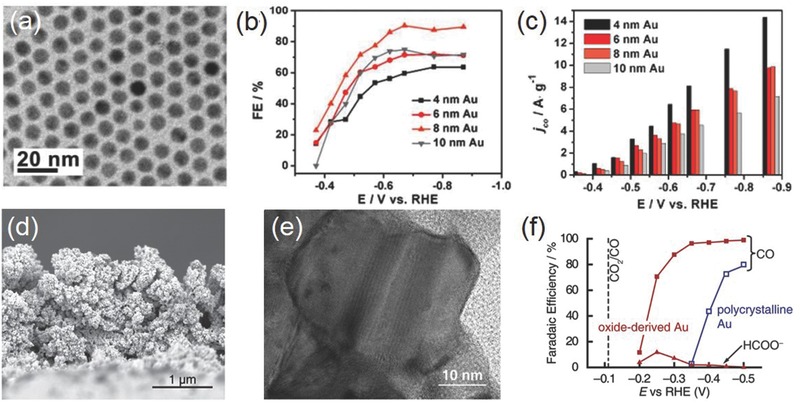
a) Transmission electron microscopy (TEM) image of 8 nm Au NPs; b) potential‐dependent Faradaic efficiency for CO on Au NPs with different sizes; c) current densities for CO formation at various potentials. Reproduced with permission.^63^ Copyright 2013, American Chemical Society. d) Cross‐sectional SEM image and e) high‐magnification TEM image of oxide‐derived Au NPs; f) Faradaic efficiency for CO and formate on oxide‐derived Au NPs in 0.5 m NaHCO_3_. Reproduced with permission.^65^ Copyright 2012, American Chemical Society.

Like Cu NPs, the reaction activity and selectivity of Au NPs strongly depends on their particle size. Smaller NPs are not necessarily more desirable for electrocatalytic CO_2_RR. Sun and co‐workers prepared monodispersed Au NPs having diameters of 4, 6, 8, and 10 nm and found that the 8 nm Au NPs exhibited the optimal activity and Faradaic efficiency for CO (90% at −0.67 V vs RHE) (**Figure** **6**a–c).^63^ DFT calculations disclosed that edge sites on the Au NP surface facilitated CO formation by stabilizing key intermediates such as COOH while the corner sites were active for HER owing to their increased affinity toward H. The highest selectivity observed with 8 nm Au NPs was attributed to their optimal ratio of edge sites over corner sites. Similar size dependence was also reported by Cuenya and co‐workers for Au NPs of 1–8 nm.^64^ As the NP size decreased, the authors observed a dramatic increase in current density and a significant decline in CO selectivity. It was rationalized by the increasing low‐coordinated sites that were suggested to favor HER over CO_2_ reduction.

The CO_2_RR activity of Au can be dramatically boosted by properly engineering its surface defects. Kanan and co‐workers prepared oxide‐derived Au by simply applying a periodic square‐wave potential routine on Au electrodes and then electrochemically reducing thick Au oxide films (Figure 6d–f).^65^ It was suggested that unique metastable structures were resulted from the reduction of the oxide film. This oxide‐derived Au exhibited selective CO_2_ reduction to CO in aqueous solution at overpotentials as low as 140 mV, high CO Faradaic efficiency (>80% at potentials more cathodic than −0.3 V), and stability for at least 8 h. Its exceptional activity was attributed to the surface metastable structures stabilizing the CO_2_^•−^ intermediate and accelerating the reduction process, although no detail was given on the nature of these metastable sites. The same research group later reported that grain boundaries were beneficial toward the CO_2_RR activity of Au.^66^ They uncovered that there was a linear correlation between the grain boundary surface density and specific activity for CO_2_ reduction on vapor deposited Au NPs on carbon nanotubes.

In addition to NPs, Au‐based materials with other nanoscale morphology have also been actively pursued for CO_2_RR. There is an enriched library of Au nanostructures with the precisely controlled geometry that provides the necessary material basis for electrochemical studies. For example, Sun and co‐workers prepared ultrathin Au nanowires by seed‐mediated growth method.^67^ The product was featured with dominant surface edge sites, and capable of highly efficient CO_2_ reduction to CO with low onset potential of −0.2 V versus RHE, high Faradaic efficiency of 94%, and mass activity of 1.84 A g^−1^ Au at −0.35 V. Stable Au concave rhombic dodecahedra were prepared by Nam and co‐workers by adding 4‐aminothiophenol during seed‐mediated growth to bind and stabilize various high‐index crystal planes such as (331), (221) and (553).^68^ Electrochemical measurement showed that concave rhombic dodecahedra exhibited improved CO selectivity (>80% between −0.8 and −0.4 V) and mass activity compared to Au films, cubes, or rhombic dodecahedra.

#### 3.1.3 Silver

Ag is the second noble metal that can enable the highly selective reduction of CO_2_ to CO, but it is relatively less active than Au due to its intrinsically weaker binding toward reaction intermediates.^45^, ^62^, ^69^ For bulk Ag metal, Hori and co‐workers found that the electrocatalytic activity of CO_2_ reduction to CO was substantially faster on atomically stepped Ag (110) than that on flat Ag (100) or Ag (111).^70^ Such a dependence now is understood as Ag (110) binds COOH more strongly than other facets.^69^ In addition to CO and H_2_ as the major products, Jaramillo and co‐workers identified four minor reduction products including formate, methane, methanol, and ethanol.^71^ They found that H_2_ was dominant at low and high overpotentials, while CO overtook H_2_ in the medium overpotential region. Those minor products, on the other hand, only appeared at very negative potentials (←1.2 V vs RHE) since they involved the further reduction of CO intermediate that was only weakly adsorbed on Ag.

**Figure 7:**
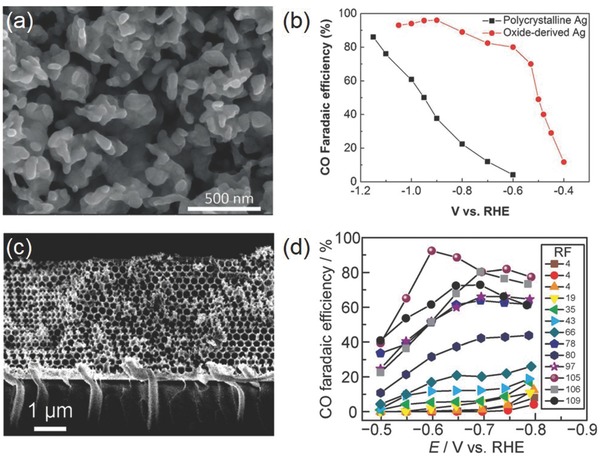
a) SEM image of oxide‐derived Ag; b) Faradaic efficiency for CO on polycrystalline Ag and oxide‐derived Ag. Reproduced with permission.^73^ c) Cross‐sectional SEM image of an Ag‐IO film; d) potential‐dependent Faradaic efficiency for CO on Ag films with varying roughness factors. Reproduced with permission.^75^

Nanostructured Ag is considerably more attractive than bulk Ag metal for CO_2_RR. Studies on Ag NPs by Masel and co‐workers showed an increasing CO_2_RR current density as their size decreased from 200 to 5 nm.^72^ However, the current density significantly dropped if the size further decreased to 1 nm. The authors interpreted their results as a consequence of variations in the binding energy of intermediates when the particle size decreased. Nevertheless, a different view was held by Jung and co‐workers proposing that the highest CO_2_RR activity for 5 nm Ag NPs was due to the optimal ratio of edge sites that were calculated to be the most CO_2_RR active—like in the case of Au NPs.^69^ Porous Ag also represents a popular choice of materials. Smith and co‐workers reported that the oxide‐derived Ag electrode from the anodization of Ag foil in alkaline solutions had a highly porous structure (**Figure** **7**a,b).^73^ It attained ≈80% Faradaic efficiency for reducing CO_2_ to CO at a moderate overpotential of 0.49 V, much enhanced than untreated polycrystalline Ag (≈4%) under identical conditions. This improvement was likely correlated with the nanostructured surface populated with highly active sites for stabilizing COOH intermediate as well as a high local pH arising from porosity‐induced transport limitation. A nanoporous silver was synthesized by Jiao and co‐workers from two‐step dealloying of an Ag‐Al precursor.^74^ Electrochemical measurements demonstrated that it was capable of reducing CO_2_ to CO with ≈92% selectivity at a rate >3000 times higher than bulk Ag under moderate overpotentials (<0.5 V). These authors likewise attributed this superior activity to the curved surface having a high density of step sites with possibly higher‐index facets that stabilized CO_2_^•−^ intermediate, therefore lowering the thermodynamic barrier for its reduction. More recently, Surendranath and co‐workers developed mesostructured Ag inverse opal electrodes and showed that with the increasing electrode mesostructural roughness, its specific activity for CO_2_ reduction to CO systematically rose by threefold and that for catalyzed HER systematically declined by tenfold (Figure 7c,d).^75^ They suggested that the mesostructured‐induced transport limitation was the primary cause for the possibility of tuning both catalyst selectivity and efficiency.

#### 3.1.4 Tin

**Figure 8:**
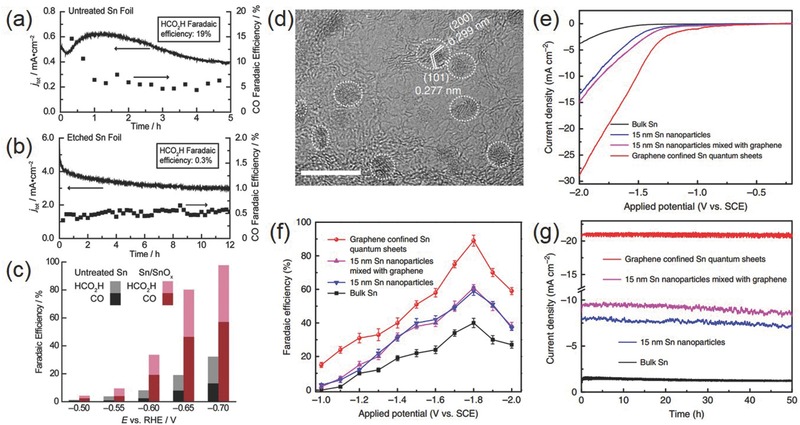
a,b) Change of the total current density and CO Faradaic efficiency with time on a) untreated Sn and b) etched Sn at −0.7 V versus RHE in 0.5 m NaHCO_3_; c) their potential‐dependent Faradaic efficiency for CO and formic acid. Reproduced with permission.^65^ Copyright 2012, American Chemistry Society. d) High‐magnification TEM image of Sn quantum sheets confined in graphene; e) polarization curves, f) potential‐dependent Faradaic efficiency for formate, and g) chronoamperometry stability at −1.8 V versus SCE on Sn quantum sheets confined in graphene as well as several control samples in 0.1 m NaHCO_3_ aqueous solution. Reproduced with permission.^79^ Copyright 2016, Nature Publishing Group.

According to Hori et al., bulk Sn metal electrode requires large overpotential (>0.86 V) in order to generate moderate current density (5.0 mA cm^−2^) for CO_2_ reduction to formic acid with the Faradaic efficiency of 88.4%.^45^ It is worth noting that in contrast to Au and Ag, the surface of Sn (particularly nanostructured Sn) rapidly gets oxidized upon exposure to air. This surface oxide layer may not be fully reduced even during CO_2_RR electrocatalysis, and therefore would greatly influence the electrochemical process. In order to elucidate the possible effect of the surface oxide layer, Kanan and co‐workers compared the activity of an Sn electrode with native SnO*_x_* layer and an electrode etched to expose metallic Sn^0^ surface (**Figure** **8**a–c).^65^ The latter exhibited higher overall current densities but almost exclusively H_2_ over the entire potentials range examined. It was proposed that SnO*_x_* directly participated in CO_2_RR pathway by stabilizing CO_2_^•−^ intermediate, otherwise HER dominated because the electron transfer to CO_2_ was prohibitively slow on metallic Sn. Zhou and co‐workers further explored the dependence of CO_2_RR selectivity on the surface SnO*_x_* thickness and uncovered that the electrode with an initial SnO*_x_* thickness of ≈3.5 nm delivered the maximum Faradaic efficiency of 64% for formate while CO production reached its highest Faradaic efficiency of 35% with an initial SnO*_x_* layer thickness of 7.0 nm.^76^ Further thickening the oxide layer resulted in a heightened HER rate. Even though the surface oxide layer cannot be not fully reduced during CO_2_RR, their partial reduction may result in the formation of a high density of grain boundaries that are usually beneficial to CO_2_RR as demonstrated by Spurgeon and co‐workers.^77^

Hybridizing Sn or SnO*_x_* nanoparticles with conductive carbon materials is proven a successful strategy to promote the CO_2_RR performance. Meyer and co‐workers prepared ≈5 nm SnO_2_ NPs uniformly deposited on graphene.^78^ CO_2_ reduction to formate was found to take place at overpotentials as low as ≈340 mV and with Faradaic efficiency of >93% at −1.8 V versus SHE in 0.1 m NaHCO_3_ aqueous solutions. This notable activity was believed to arise from the compromise between the adsorption strength of CO_2_^•−^ and its subsequent kinetic activation on the nanoscale Sn surface as well as the electronic interactions between the graphene support and metal NPs. Xie and co‐workers recently showed that metallic Sn quantum sheets confined in graphene displayed a large current density of 21.1 mA cm^−2^, Faradaic efficiency of ≈90% and great stability for over 50 h at −1.8 V versus saturated calomel electrode (SCE) for selective CO_2_ reduction to formate (Figure 8d–g).^79^ It was suggested as the collective result of the highly conductive graphene support facilitating the rate‐determining electron transfer step from CO_2_ to CO_2_^•−^ and the graphene confined Sn quantum sheets stabilizing the CO_2_^•−^ radical with its low‐coordinated sites.

#### 3.1.5 Other Metals

There are many other metals investigated for CO_2_RR electrocatalysis. Pd can reduce CO_2_ to CO but its Faradaic efficiency is generally much lower than that on Au or Ag due to the competing HER.^45^, ^80^ One possible remedy to this poor selectivity is to control its particle size below 5 nm for optimal edge and corner site density.^81^ Occasionally, formate is identified as the main reduction product on Pd as probably mediated by the surface PdH_x_.^82^ There are also a handful of reports about Zn‐, In‐, or Bi‐based materials, mostly prepared from electrodeposition, for selectively reducing CO_2_ to CO.^83^, ^84^, ^85^, ^86^, ^87^, ^88^, ^89^ Even though Co is commonly regarded as HER‐active, partially oxidized Co ultrathin layers surprisingly reduce CO_2_ to formate at a very high Faradaic efficiency.^90^, ^91^ Besides pure metals, alloying of two different metals is explored so as to tune the binding strength of key intermediates through geometric and electronic effects, and therefore optimize the CO_2_RR reaction activity and selectivity. Thus far, most research attention has been focused on Cu‐based alloys, such as Cu–Au, Cu–Pd, Cu–In and Cu–Pt and Cu–Sn, and some showed very exciting results.^92^, ^93^, ^94^, ^95^, ^96^ It can be reasonably expected that the alloying strategy would soon be expanded to other combination for better possibilities.

### 3.2 Metal Chalcogenides

Layered transition metal dichalcogenides (TMD) such as MoS_2_, MoSe_2_, and WS_2_ have been widely investigated as the HER electrocatalysts.^97^ However, it is not until recent years that their potential in CO_2_RR starts to be unveiled. Nørskov and co‐workers used DFT calculations to explore the binding properties of CO_2_RR intermediates (COOH, CHO, and CO) on MoS_2_ and MoSe_2_ edges.^98^, ^99^ COOH and CHO were found to prefer bridging S or Se atoms, while CO was selectively adsorbed on the metal atom. These authors argued that in this way, the active edges may break the scaling relations observed between intermediates on transition metals, making them potentially more attractive for CO_2_RR than even the best transition metals. Because TMD materials are excellent HER electrocatalysts, CO_2_RR of TMD materials usually have to be carried out in mixture solution of ionic liquid (e.g., EMIM‐BF_4_) and water in order to suppress HER. Some ionic liquids are reported to form a stable complex with the CO_2_^•−^ intermediate so that they can lower the activation energy barrier for effective CO_2_ reduction.^100^

**Figure 9:**
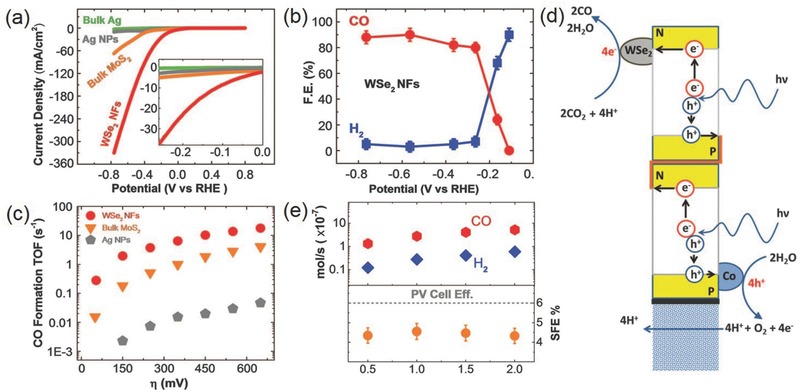
a) CV curves of WSe_2_ NFs, bulk MoS_2_, Ag NPs, and bulk Ag in CO_2_‐saturated EMIM‐BF_4_/H_2_O solution; b) potential‐dependent Faradaic efficiency for CO and H_2_ on WSe_2_ NFs; c) CO formation TOF of WSe_2_ NFs, bulk MoS_2_, and Ag NPs; d) schematic showing an artificial leaf with WSe_2_ cocatalyst for reducing CO_2_ to CO under light illumination. e) Product formation rates under different light illumination intensities using the WSe_2_/IL cocatalyst system. Reproduced with permission.^102^ Copyright 2016, American Association for the Advancement of Science.

In 2014, Salehi‐Khojin and co‐workers first experimentally demonstrated bulk MoS_2_ as a highly efficient electrocatalyst for selectively reducing CO_2_ to CO with a small overpotential of 54 mV in a mixture of 96 mol% water and 4 mol% EMIM‐BF_4_.^101^ An impressive cathodic current density of ≈65 mA cm^−2^ and a CO Faradaic efficient of 98% were delivered at η = 0.65 V, much improved than Au and Ag NPs. In a follow‐up study, the same research group compared the CO_2_RR performance of four different TMD materials (MoS_2_, MoSe_2_, WS_2_, and WSe_2_) with similar sizes and identified that WSe_2_ nanoflakes were the most active.^102^ At η = 54 mV, it exhibited an exceptional current density of ≈19 mA cm^−2^, CO Faradaic efficiency of 24%, and TOF of 0.28 s^−1^ in 50:50 vol% ionic liquid/water electrolyte; at η = 0.65 V, the recorded current density for WSe_2_ reached 330 mA cm^−2^ with a Faradaic efficiency of ≈85%—an unprecedented activity surpassing any other known CO_2_RR electrocatalysts (**Figure** **9**). DFT calculations revealed that unlike transition metal surfaces, COOH formation on TMD edges was exergonic, and CO was also stabilized, indicating the formation of CO from CO_2_ was kinetically favorable. The authors also built a proof‐of‐concept artificial leaf by coupling Si photovoltaic cells with oxygen evolution reaction (OER) and CO_2_RR electrocatalysts in a wireless configuration and achieved concurrent production of CO and oxygen under light illumination. Moreover, proper doping of TMD materials may further push their performance to the limit. 5% Nb doped vertically aligned MoS_2_ was found to exhibit the smallest onset overpotential of 31 mV, and one order of magnitude higher CO formation TOF than pristine MoS_2_ within an overpotential range of 50–150 mV.^103^ The presence of Nb was suggested to facilitate the rapid release of CO from the TMD edge. Despite these exciting progresses on this family of materials, it should be noted that their excellent CO_2_RR activities are yet to be confirmed by other research groups.

CO_2_ may also be reduced to liquid products on metal chalcogenides. Han and co‐workers recently showed that Mo‐Bi sulfide could reduce CO_2_ to methanol in 0.5 m BMIM‐BF_4_ in acetonitrile.^104^ Its Faradaic efficiency reached a record value of 71.2% with a current density of 12.1 mA cm^−2^ at −0.7 V versus SHE. The synergy between Mo and Bi was speculated as the main origin of its high activity, where Bi favored the transformation of CO_2_ to CO, and Mo promoted the further hydrogenation of CO to methanol.

### 3.3 Carbonaceous Materials

Carbonaceous materials are also applied for CO_2_RR electrocatalysis by virtue of their good electrical conductivity, low cost, chemical stability and usually large surface area. However, pristine carbonaceous materials are generally inert and have negligible activity for CO_2_RR because they can hardly activate CO_2_ molecule or adsorb CO_2_^•−^ intermediate.^105^, ^106^ It is a different scenario when carbonaceous materials are properly doped with heteroatoms (e.g., N, B, P, and S). Doping introduces structural defects or induces charge/spin densities on the adjacent carbon atoms, therefore significantly altering the interaction between carbonaceous materials and CO_2_ or reaction intermediates.

Up to now, various forms of heteroatom functionalized carbonaceous materials including carbon nanotubes, nanofibers, graphene sheets, graphene quantum dots and nanodiamonds have been pursued and investigated in CO_2_ reduction. Salehi‐Khojin and co‐workers prepared N‐doped carbon nanofibers (CNFs) from pyrolyzing electrospun polyacrylonitrile precursor.^107^ It exhibited a small onset overpotential of 170 mV for selectively reducing CO_2_ to CO in EMIM‐BF_4_ and delivered more than an order of magnitude higher current density than bulk Ag under similar experimental conditions. The electrocatalytically active sites were proposed to be highly positive carbon atoms adjacent to electronegative N dopants. Liquid C_1_ products are identified from the CO_2_RR on some carbonaceous materials. For example, Einaga and co‐workers discovered the sp^3^‐bonded carbon in B‐doped diamond (BDD) electrode selectively reduced CO_2_ to formaldehyde with Faradaic efficiency up to 74% in methanol or seawater, even though the current density was considerably low (<0.3 mA cm^−2^).^108^ Meyer and co‐workers achieved Faradaic efficiency for formate production of 87% with current densities of 9.5 mA cm^−2^ at −1.8 V versus SCE on polyethylenimine‐enhanced N‐doped carbon nanotubes in 0.1 m KHCO_3_ solution.^105^ In addition, C_2_–C_3_ products are also obtained. Quan and co‐workers observed that N‐doped nanodiamond (NDD) demonstrated Faradaic efficiency of ≈77% toward acetate production and ≈15% toward formate formation at a potential range of −0.8–−1 V versus RHE.^109^ It was the first example of CO_2_RR electrocatalysts other than Cu that were able to convert CO_2_ to a C_2_ product with such high selectivity. Using N‐doped graphene quantum dots, Ajayan and co‐workers reported that CO_2_ could be reduced into multicarbon hydrocarbons and oxygenates with total Faradaic efficiency of CO_2_ reduction of up to 90% and selectivity for ethylene and ethanol reaching 45%.^110^ The C_2_ and C_3_ product distribution and production rate was comparable to those obtained with Cu‐based electrocatalysts.

### 3.4 General Material Designing Strategies

Based on the above discussion, a few general material designing strategies can be identified across various CO_2_RR electrocatalysts and are summarized as follows. *(1) Nanostructuring*: Nanostructuring so far is the most common and popular approach to promote the performance of catalyst materials.^111^, ^112^ When the size of catalyst particles is reduced down to the nanoscale, their surface areas are dramatically enhanced, which more than often leads to heightened activities. However, one thing worth noting here is that unlike most other catalytic reactions, smaller particle size is not always beneficial to CO_2_RR as we have seen for Cu, Au, and Ag metals. This is because too small particles have an increasing number of low‐coordinated sites that favor HER over CO_2_RR. Apart from the particle size, we now are able to precisely tune the morphology of catalyst materials at the atomic scale with the assistance of nanotechnology, and many delicate nanostructures such as nanocubes, nanosheets, nanowires and so on can be readily prepared by carefully controlling the reaction conditions. This brings us the possibility to explore the morphology dependence of their catalytic activity and selectivity and to gain deeper insight into their structure–property correlation. Moreover, nanostructuring may also introduce structural defects such as vacancies and grain boundaries with local chemical environment and electronic structure distinct from the bulk. They may serve as the CO_2_RR active sites with unexpected performance. *(2) Doping or alloying*: Doping or alloying with foreign atoms is an effective way to adjust the binding energy of CO_2_ and key reduction intermediates on catalyst surfaces, and thus has the great potential to modulate the CO_2_RR activity and selectivity. The proper choice of dopant types, doping level or alloying composition can be guided by DFT‐based computations. Cu is the only elemental metal capable of producing C_1_–C_3_ hydrocarbons at significant rates due to its suitable binding energy toward the CO intermediate. Stronger CO binding (on catalysts such as Fe, Ni, Co, and Pt) encourages HER, whereas weaker CO binding (on catalysts such as Sn, Pb and Hg) favors formate formation.^113^, ^114^ By combing metals having strong CO binding property with metals having weak CO binding property, one may arrive at an intermediate CO‐catalyst interaction that promotes the formation of higher‐value reduction products. *(3) Hybridization with carbon*: Electrocatalysis relies on the conduction of electricity to drive catalytic reactions. A high surface area and conductive carbon support (such as graphene, carbon nanotubes, carbon fibers, and so on) would not only greatly benefit the electron transport to and from electrocatalysts but also improve their dispersion. The synergy between the support and the electrocatalyst may also afford an unexpected gain in activity and selectivity. However, care should be taken when designing such hybrid electrocatalysts since many carbon materials may contain metal impurities (such as Fe, Co, and Mo that are often used as the catalyst seed for the growth of carbon nanotubes) that promote the HER side reaction.

## 4 Photocatalytic Materials for CO_2_ Reduction

In addition to electrical energy, light can also provide the necessary energy stimulus in order to break the C=O bond in CO_2_. Since the first report about the photocatalytic application of TiO_2_ by Fujishima and Honda in 1972,^115^ there have been increasing research activities on semiconductor photocatalytic materials, initially for water splitting and more recently for CO_2_ reduction. A number of strategies have been developed and implemented by engineering their structures at different scales so as to promote their efficiency in light absorption, charge separation and interfacial charge transfer. In this part, we aim to review different photocatalysts (**Table** **3**) and their design strategies and to highlight our current understanding of these complex systems.

**Table 3:** Summary of CO_2_ reduction photocatalysts from recent literature

Photocatalyst	Cocatalyst	Light source	Experimental condition	Main products and highest yield	Reference
Anatase TiO_2_ (0.1 g)	–	300 W Xe lamp	CO_2_ and H_2_O vapor	CH_4_ 1.35 µmol h^−1^ g^−1^	199
TiO_2_ single crystals (0.02 g)	Pt	400 W Xe lamp	CO_2_ and H_2_O vapor	CH_4_ 1361 µmol h^−1^ g^−1^ (QE (CH_4_) = 2.41%).	211
Commercial P25 (1.25 cm^2^)	Au–Cu nanoalloys	Sun simulated light (1000 W Xe lamp)	CO_2_ and H_2_O vapor	CH_4_ 2300 µmol h^−1^ g^−1^	212
Rutile TiO_2_ modified anatase TiO_2_ nanorods (0.1 g)	–	300 W Hg lamp	CO_2_ and H_2_O vapor	CH_4_ 2.5 µmol h^−1^ g^−1^	230
Degussa P25 (0.05 g)	Cu2+, Cu+, and Cu+/Cu0	150W solar simulator	CO_2_ and H_2_O vapor	CO 25 µmol g^−1^, CH_4_ 25 µmol g^−1^	231
Defective TiO_2_ (anatase, rutile, and brookite) (0.1 g)	–	A 150 W solar simulator	CO_2_ and H_2_O vapor	CH_4_ 17 µmol g^−1^	120
TiO_2_ (0.1 g)	Ag	8 W Hg lamp	CO_2_ bubbled solution	CH_4_ 9 µmol g^−1^, CH_3_OH 1.8 µmol g^−1^	232
Anatase TiO_2_ nanosheets exposed with 95% of {100} facets (0.04 g)	–	300 W Xe lamp	CO_2_ and H_2_O vapor	CH_4_ 5.8 ppm g^−1^ h^−1^	233
Codoped TiO_2_ (0.1 g)	–	300 W Xe lamp (λ > 420 nm)	CO_2_ and H_2_O vapor	CO 1.9, CH_4_ 0.09 µmol h^−1^ g^−1^	174
TiO_2_ (0.4 g)	Ag	500 W Xe lamp (λ > 420 nm)	CO_2_ saturated H_2_O	CH_3_OH 400 µmol g^−1^ (3 h)	234
Defective single‐unit‐cell BiVO_4_ layers (0.2 g)	–	300 W Xe lamp (AM1.5)	CO_2_ saturated water	Methanol 398.3 µmol h^−1^ g^−1^,	131
BiVO_4_ and CuGaS_2_ (0.05 g)	CoO*_x_*/Pt	300 W Xe lamp (λ > 420 nm)	CO_2_ saturated K_2_SO_3_ solution	CO 6 µmol h^−1^ g^−1^	220
ZnAl_2_O_4_‐modified ZnGaNO (0.1 g)	Pt	300 W Xe‐lamp (λ > 420 nm)	CO_2_ and H_2_O vapor	CH_4_ 9.2 µmol h^−1^ g^−1^	148
Ni doped anatase TiO_2_ (0.5 g)	–	18 W cm^−2^ Hg lamp	CO_2_ saturated water	CO 14 µmol g^−1^	235
AgBr/TiO_2_ (0.5 g)	–	150 W Xe lamp (λ > 420 nm)	CO_2_ saturated KOH solution	CH_4_ 128.56, CH_3_OH 77.87, C_2_H_5_OH 13.28, CO 32.14 mol g^−1^,	236
Titanate nanosheet‐assembled Yolk@Shell Microspheres (0.1 g)	–	150 W Xe lamp (λ > 420 nm)	CO_2_ saturated water (NaHCO_3_+HCl)	CH_3_OH 2.1 µmol h^−1^ g^−1^	185
Graphene‐Ti_0.91_O_2_ hollow spheres (0.01 g)	–	300 W Xe‐lamp	CO_2_ and H_2_O vapor	CO 9, CH_4_ 1 µmol h^−1^ g^−1^	184
Nifion coated TiO_2_ particles (unspecified)	Pd (1 wt%)	300 W Xe‐lamp	CO_2_ saturated Na_2_CO_3_	CH_4_ 6, C_2_H_6_ 5 µmol h^−1^	237
Degussa P25 (unspecified)	Pt‐Cu_2_O	200 W Xe lamp (λ = 320–780 nm).	CO_2_ and H_2_O vapor	CH_4_ 33, CO 8.3, H_2_ 25 µmol h^−1^ g^−1^	238
Sandwich‐like graphene‐TiO_2_ hybrid sheets (0.1 g)	–	300 W Xe‐lamp	CO_2_ and H_2_O vapor	C_2_H_6_ 16.8, CH_4_ 8 µmol h^−1^ g^−1^	239
Porous silica supported Cu/TiO_2_ catalysts (0.1 g)	–	Xe‐lamp 2.4 mW cm^−2^	CO_2_ and H_2_O vapor	CO 45, CH4 13.2 µmol h^−1^ g^−1^ (QE (CO_2_) = 1.41%)	240
SrTiO_3_/TiO_2_ coaxial nanotube arrays (0.005 g)	Au–Cu Alloy NPs	300 W Xe lamp	CO_2_ bubbled N_2_H_4_·H_2_O solution	CO 165 ppm cm^−1^ h^−1^	241
TiO_2_/ZnO powder (0.1 g)	–	300 W Xe lamp (60 mW m^−2^)	CO_2_ and H_2_O vapor	55 µmol h^−1^ g^−1^	242
In doped anatase TiO_2_	–	400 W Hg lamp	CO_2_, He, and H_2_O vapor	CH_4_ 243.75, CO 81.25 µmol h^−1^ g^−1^	243
Anatase TiO_2_ single crystals with {101} facets (0.1 g)	RuO_2_	300 W Xe lamp	CO_2_ and H_2_O vapor	CH_4_ 1.8, H_2_ 80, O_2_ 15 µmol h^−1^ g^−1^	244
Graphene–WO_3_ nanobelt (0.1 g)	–	300 W Xe lamp (λ > 400 nm)	CO_2_ and H_2_O vapor	CH_4_ 0.1, O_2_ 3.5 µmol h^−1^	245
WO_3_ (0.1 g)	–	300 W Xe lamp	CO_2_ and H_2_O vapor	CH_4_ 16 µmol g^−1^	126
Nb_3_O_8_‐nanosheets (unspecified)	amorphous Cu clusters	Hg‐Xe lamp (240–300 nm)	0.5 m KHCO_3_ aqueous solution (PH = 12)	CO 0.07 µmol h^−1^	246
Defective single‐unit‐cell BiVO_4_ (0.2 g)	–	300 W Xe lamp	CO_2_ and H_2_ vapor	CH_3_OH 398.3 µmol g^−1^ h^−1^ (AQE = 5.95%, 350 nm)	131
Single unit cell Bi_2_WO_6_ (0.2 g)	–	300 W Xe lamp (100 mW cm^−2^)	CO_2_ and H_2_O vapor	Methanol 502 µmol g^−1^ h^−1^	183
NaTaO_3_ (0.07 g)	Pt or Ru	300 W Xe lamp (λ > 200 nm)	CO_2_, H_2_O, and H_2_ vapor	Pt/NaTaO_3_ (CO 139.1 µmol g^−1^ h^−1^)	247
Single‐crystalline Zn_2_GeO_4_ nanobelts	RuO_2_/Pt	300 W Xe lamp	CO_2_ and H_2_O vapor	CH_4_ 6 µmol h^−1^ g^−1^	134
Porous Ga_2_O_3_ (0.05 g)	–	300 W Xe lamp	CO_2_ and H_2_O vapor	CH_4_ (170 ppm) CO quantum yield 3.993%	248
Mesoporous ZnGa_2_O_4_ (0.1 g)	RuO_2_	300 W Xe lamp	CO_2_ and H_2_O vapor		249
In_2_O_3_ nanobelts coated with carbon layer (0.2 g)	Pt	300 W Xe lamp	CO_2_ saturated H_2_O (10% triethanolamine (TEOA))	CO, 126.6 CH_4_ µmol h^−1^	250
Cu_2_O/reduced graphene oxide (RGO) (0.5 g)	–	300 W Xe lamp	CO_2_ and H_2_O vapor	CO 50 ppm h^−1^ g^−1^	251
Nitrogen doped ZnO (0.01 g)	Cu	8 W fluorescent tube	CO_2_ and H_2_O vapor molar ratio of 6.7 (CO_2_:H_2_O)	CO (0.73 µmol h^−1^ g^−1^), CH3OH, CH_4_, H_2_	252
CeO_2_ (0.1 g)	Pt	300 W Xe lamp	CO_2_ and H_2_O vapor	CH_4_ 1.12 µmol h^−1^ g^−1^.	253
Ni/SiO_2_·Al_2_O_3_ (1.5 cm^2^)	–	Solar simulator	CO_2_, N_2_, H_2_ vapor	CH_4_ (highest selectivity 99.9%), CO, C_2_H_6_,	254
Co_3_O_4_ with exposed {112} facets ([Ru(bpy)_3_]Cl_2_ as a photosensitizer) (0.01 g)	–	300 W Xe lamp (λ > 420 nm)	CO_2_ saturated acetonitrile/TEOA/H_2_O (3:1:1) solution	CO 1297, H_2_ 502 µmol g^−1^ h^−1^	255
Graphene oxide (GO)‐CdS nanorods (0.01 g)	–	300 W Xe lamp (λ > 420 nm) (150 mW cm^−2^)	CO_2_ and H_2_O vapor	CH_4_ 2.51 µmol h^−1^ g^−1^	256
Cu_2_S nanorod (unspecified)	Pt	450 W Xe lamp	1 m Na_2_CO_3_	CO 3.02, CH_4_ 0.13 µmol h^−1^ g^−1^.	257
Bi_2_S_3_ (0.01 g)	–	250 W Hg lamp	CO_2_ saturated methanol	HCOOH 700 µmol g^−1^ (4 h)	139
GaN nanowire arrays (3.5 cm^2^)	Rh/Cr_2_O_3_	300 W Xe lamp	CO_2_ and H_2_O vapor	CH_4_ ≈3.5 µmol g^−1^ h^−1^ in 24 h.	144
MgAl layered double oxide (LDO) grafted TiO_2_ (0.1 g)	–	450 W Xe lamp (λ > 400 nm)	CO_2_ and H_2_O vapor (reaction temperature at 150 °C)	CO 1 µmol h^−1^ g^−1^	258
Mg doped InGaN/GaN nanowire (3 cm^2^)	Pt	300 W Xe lamp (AM1.5 G filter)	CO_2_ and H_2_ vapor (1:4)	CH_3_OH 500 µmol g^−1^ h^−1^	145
ZnCu–M(III) (M = Al, Ga) LDH (0.1 g)	–	500 W Xe lamp	CO_2_ and H_2_ vapor	CH_3_OH 0.49, CO 0.62 µmol h^−1^ g^−1^	155
Ni/Mg/Zn‐Ga/Al/In‐LDH (0.1 g)	–	200 W Hg‐Xe lamp	CO_2_ and H_2_ vapor	CO 3.21, O_2_ 17 µmol h^−1^ g^−1^	158
MgAl‐LDH (unspecified)	Pd	500 W Xe lamp	CO_2_ saturated water	CH_4_ 3.7 µmol	259
Defect rich Zn‐Al LDH nanosheet (0.1 g)	–	300 W Xe lamp	CO_2_ and H_2_ vapor	CO 8 µmol h^−1^ g^−1^	159
C_3_N_4_ (0.008 g)	C_3_N_4_	400 W Hg lamp	Solution of 4:1 v/v solvent (MeCN, N,N′‐dimethylacetamide (DMA), MeOH, or water):TEOA	HCOOH, TON(>1000), AQY (5.7%, 400 nm)	172
Graphene–g‐C_3_N_4_ hybrid (unspecified)	–	15 W daylight bulb (8.5 mW cm^−2^)	CO_2_ and H_2_O vapor	CH_4_ 5.87 µmol g^−1^	260

### 4.1 Compositions of Semiconductor Photocatalysts

#### 4.1.1 Metal Oxide

**Figure 10:**
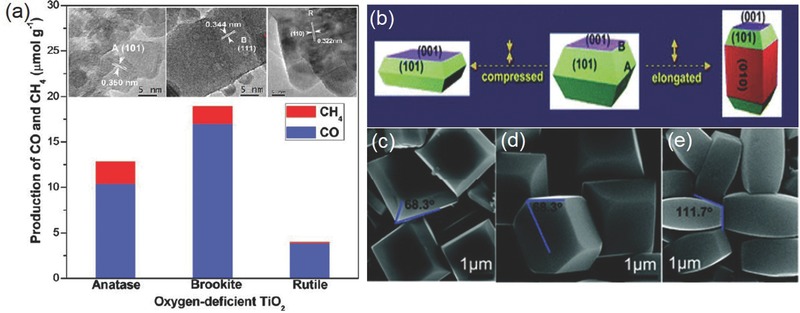
a) Production rates of CO and CH_4_ on three TiO_2_ nanocrystal polymorphs (anatase, rutile, and brookite). Reproduced with permission.^120^ Copyright 2012, American Chemical Society. b) Schematic of anatase TiO_2_ with different percentages of {101}, {001}, and {010} facets and c–e) SEM images of corresponding synthetic products. Reproduced with permission.^122^

Metal oxides are a very common type of photocatalyst materials for CO_2_ reduction. A large number of them consist of transition metal cations (e.g., Ti^4+^, Zr^4+^, Nb^5+^, Ta^5+^, W^6+^, and Mo^6+^) with the *d* ^0^ configuration. Their conduction bands are composed of vacant metal *d* orbitals and usually more negative than 0 V, while their valence bands are composed of O 2p orbitals and usually more positive than 3 eV.^116^ The band structure of these metal oxides can generally enable the simultaneous CO_2_ reduction and water oxidation, but their wide band gap more than often restricts the utilization of solar spectrum only within the UV region.^117^, ^118^ TiO_2_ is the most representative and well‐studied *d* ^0^ metal oxide semiconductor photocatalyst with the advantages of low cost, low toxicity, and chemical stability.^119^ Among its three polymorphs that naturally exist, the anatase form of TiO_2_ receives wide attention and is shown to be highly active in photocatalytic CO_2_ reduction.^16^ Comparatively, rutile is less active due to its fast charge recombination, and brookite, on the other hand, is rarely investigated for photocatalysis probably due to the past difficulty in obtaining phase‐pure brookite. However, increasing evidence now suggests that pure brookite has a high activity for photocatalytic CO_2_ reduction, and oxygen‐deficient brookite is even more appealing than anatase due to its enhanced interaction with and charge transfer to the CO_2_ molecule (**Figure** **10**a).^120^, ^121^ The photocatalytic activity of TiO_2_ also depends on exposed crystal facets. Even though thermodynamically less stable than other low‐index facets such as {101} and {001}, the {010} facet of anatase TiO_2_ is shown to be more active (Figure 10b), presumably benefited from its more favorable surface atomic structure having 100% five‐coordinated Ti atoms and slightly more negative conduction band.^122^, ^123^, ^124^ DFT calculations also predict that the interaction of CO_2_ with {010} was stronger than its interactions with {101} or {001}.^125^ Apart from TiO_2_, other transition metal oxides such as WO_3_ and ZrO_2_ as well as oxysalts such as titanates (ATiO_3_, A = Na, Sr, Ca, or Pb), tantalates (ATaO_3_, A = Li, Na, or K), niobates (ANbO_3_, A = Na, K), tungstate (Bi_2_WO_6_), and vanadates (BiVO_4_, InVO_4_, Na_2_V_6_O_16_·*x*H_2_O) have also been explored in photocatalytic CO_2_ reduction.^126^, ^127^, ^128^, ^129^, ^130^, ^131^, ^132^ Some of them show considerable visible light activity. Furthermore, many main group metal oxides with metal cations (e.g., In^3+^, Ga^3+^, Ge^4+^, Sn^4+^, and Sb^5+^) in the *d* ^10^ configuration are also photocatalytically active. Their conduction bands consist of hybridized sp orbitals with large dispersion and are able to provide photogenerated electrons with high reducing power.^116^, ^118^ Unfortunately, their higher conduction bands translate to further broadened band gaps. For example, two popular examples—Zn_2_Ga_2_O_4_ and ZnGeO_4_ have band gaps of 4.5 and 4.4 eV, respectively, making them only responsive to the deep UV light.^133^, ^134^

#### 4.1.2 Metal Sulfide

Metal sulfides represent another large group of photocatalyst materials for CO_2_ reduction. Compared to their oxide counterparts, metal sulfides possess higher valence bands mainly of the S 3p character and have narrower band gaps. It is, however, a general concern that photogenerated holes on their valance band may not be energetic enough to oxidize water and would instead result in their irreversible photocorrosion. As a result, hole scavengers are frequently added in order to extend their stability. CdS is a well‐known visible light photocatalyst having a band gap (2.4 eV) that matches well with the solar spectrum.^116^ In 1988, Eggins et al. first reported the photocatalytic performance of CdS for CO_2_ reduction under visible light, yielding formaldehyde, methanol, formate, acetate, and glyoxylate as the main products.^135^ Wang and Wang recently coupled CdS with Co‐ZIF‐9 as the cocatalyst and achieved the conversion from CO_2_ to CO with a high apparent quantum yield of 1.93% under monochromatic irradiation of 420 nm.^136^ ZnS also attracts considerable attention for photocatalytic CO_2_ reduction. Its conduction band has very high energy and extremely low redox potential (−1.8–−2.0 V vs SHE) and can enable the one‐electron reduction of CO_2_ to CO_2_^•−^ as confirmed by electron paramagnetic resonance spin‐trapping experiments.^137^ Macyk and co‐workers demonstrated that ZnS nanoparticles functionalized with Ru cocatalyst photocatalyzed CO_2_ reduction to formic acid and CO with traces of methane.^137^ The photocatalytic activity and selectivity were found to depend on nanoparticle size and solvent polarity. The same research group also showed that when coupled with the oxidation of acetylacetone by photogenerated holes from its valence band, photocatalytic CO_2_ reduction on ZnS would lead to the formation of carboxylic acids.^138^ In addition to above compounds, other sulfides and solid solutions were also investigated.^139^, ^140^, ^141^, ^142^

#### 4.1.3 Metal Nitride

Metal nitrides or oxynitrides also have narrow band gaps due to their high valence bands mainly composed of N 2p orbitals. Despite their desirable visible light absorbance, little photocatalytic activity, however, was generally observed for *d* ^0^ transition metal nitrides and oxynitrides such as Ta_3_N_5_, TaON, MTaO_2_N (M = Ca, Sr, Ba and La) probably due to their low conduction band edge. It is suggested that *d* ^10^ metal with broadly dispersed conduction bands are more promising toward photocatalytic CO_2_ reduction.^116^, ^143^ Gallium‐based nitrides are the most studied. Mi and co‐workers reported that GaN nanowires reduced CO_2_ to CH_4_ and CO at a high conversion rate using sunlight as the only energy input.^144^ Decoration of the nanowire surface with Rh/Cr_2_O_3_ or Pt cocatalyst nanoparticles enhanced the reaction rate and selectivity toward CH_4_, reaching ≈3.5 and ≈14.8 µmol g_cat_^−1^ h^−1^ for CH_4_, respectively. In their follow‐up study, these authors employed multiband InGaN/GaN nanowires to realize the rapid transformation of CO_2_ to methanol.^145^ The photocatalytic activity was further boosted with the incorporation of Mg‐dopant, which was believed to promote CO_2_ adsorption and reduce surface potential barrier based on DFT calculations. The optimal conversion rate was measured to be 0.5 mmol g_cat_^−1^ h^−1^ with high stability over 10 h under visible light illumination (>400 nm). It is worth noting that compared to sulfides, GaN is known for its improved resistance to photocorrosion so that no hole scavenger is necessary. Other nitrides and oxynitrides investigated for photocatalytic CO_2_ reduction include ZnGeNO and ZnGaNO.^146^, ^147^, ^148^

#### 4.1.4 Layered Double Hydroxide (LDH)

LDH with the general formula of [M^2+^_1−_ *_x_*M^3+^ *_x_*(OH)_2_][A*^n^* ^−^ *_x_*_/_ *_n_*·*m*H_2_O] (where M^2+^, M^3+^, and A^n−^ are divalent cation, trivalent cation, and interlayer anion, respectively) is a class of layered materials comprising positively charged metal hydroxide layers and charge‐balancing anions between the layers.^149^ Many LDH compounds (such as Ti‐based LDHs) are known to be excellent photocatalysts for water splitting.^150^, ^151^, ^152^ Their potential in photocatalytic CO_2_ reduction was first uncovered by Izumi's group in 2011.^153^ Up to now, LDHs containing Zn^2+^, Cu^2+^, Mg^2+^, and Ni^2+^ combined with Al^3+^, Ga^3+^, Cr^3+^, and In^3+^ are shown to be active for reducing CO_2_ to CO or methanol as main products.^154^ Inclusion of Cu ions within the host layers or replacing the interlayer anions with cuprous anions generally improves the selectivity for methanol over CO production.^155^ For example, a methanol selectivity as high as 68% was reported using ZnCuGa‐CO_3_ LDH.^156^ Tanak and co‐workers compared the photocatalytic activity of several different LDH materials (composed of divalent Ni/Mg/Zn and trivalent Al/Ga/In) in aqueous solution and concluded that Ni‐Al LDH had the highest activity (110.9 µmol in 8 h) with a CO selectivity of 88.4%.^157^, ^158^ Zhang and co‐workers recently showed that reducing the thickness of ZnAl LDH nanosheets dramatically enhanced their photocatalytic activity relative to their bulk counterpart, giving rise to a remarkable CO formation rate up to 7.6 µmol g^−1^ h^−1^.^159^

#### 4.1.5 Metal‐Organic Framework (MOF)

MOFs are a family of porous materials with crystalline and open structures consisting of metal ions or clusters coordinated with organic ligands.^160^ Since both the organic ligands and metal ions can be systematically varied, MOFs possess extraordinary chemical and functional versatility. Most uniquely, they may contain photosensitizers and catalytic centers in a single solid, and thereby represent promising alternatives to conventional semiconductors for photocatalysis. For example, many Ti‐based MOFs combine the photocatalytic activity of titanium oxide clusters with the light adsorbing properties of organic linkers and are photocatalytically active under UV–vis light. Li and co‐workers first reported Ti‐containing MIL‐125‐NH_2_ with the 2‐aminoterephthalate linker as the photocatalyst for CO_2_ reduction to formate under visible light irradiation.^161^ It was followed by Uribe‐Romo and co‐workers, who prepared a series of Ti‐based MOFs isoreticular to MIL‐125‐NH_2_, where the amine functionality was decorated with alkyl chains of varying length and connectivity.^162^ The authors observed that by successively increasing the alkyl substitution, resulted MOFs displayed a gradually decreased bandgap from 2.56 to 2.29 eV and increased photocatalytic reaction rates and quantum yield for reducing CO_2_ to formate. In particular, MIL‐125‐NHCyp (Cyp = cyclopentyl) exhibited the largest AQE of 1.8%, as attributed to its long‐lived excited‐state and narrow bandgap compared to the parent MIL‐125‐NH_2_.

#### 4.1.6 Metal‐Free Material

Carbonaceous materials attract growing interest for the solar fuel production in recent years. One of the good examples is graphitic carbon nitride (g‐C_3_N_4_). It has a layered structure analogous to graphite and ideally built from tri‐s‐triazine units.^163^ In 2009, Domen and co‐workers first reported its photocatalytic activity for hydrogen production under visible light.^164^ Great efforts have been invested on C_3_N_4_ to explore its potential for photocatalytic CO_2_ reduction since 2013.^165^, ^166^, ^167^, ^168^ Various reduction products such as CO, CH_4_, C_2_H_6_, HCOOH, and CH_3_OH are measured.^169^, ^170^, ^171^ Peng and co‐workers were among the first to study the photocatalytic performance of C_3_N_4_, and observed that porous C_3_N_4_ derived from urea yielded methanol and ethanol while nonporous C_3_N_4_ derived from melamine only converted CO_2_ to ethanol.^165^ Their AQE were 0.18% and 0.08% respectively. By hybridizing C_3_N_4_ with Ru complexes, Maeda and co‐workers achieved the selective conversion of CO_2_ to HCOOH.^172^ Their best photocatalyst had a TON of greater than 1000 (20 h) under visible light irradiation with an AQE of 5.7% at 400 nm, both of which were the highest values ever reported under similar conditions. Moreover, exfoliation of bulk C_3_N_4_ powder to atomic layer thick nanosheets represents an effective route to further boost its photocatalytic performance thanks to the enlarged surface area and enhanced charge separation property of nanosheets.^173^ Ye and co‐workers observed that the hybrid of exfoliated C_3_N_4_ nanosheets with a Zr‐based MOF (UiO‐66) exhibited better charge separation efficiency and prolonged lifetime of photogenerated carriers.^171^ A much higher photocatalytic activity for CO_2_ reduction to CO (9.9 µmol g^−1^ h^−1^) was accordingly measured under visible light.

### 4.2 Photocatalytic Materials Designing Strategies

#### 4.2.1 Band Structure Engineering

As mentioned above, the band structure of a semiconductor photocatalyst determines its capability to absorb light and energize surface redox reactions. Unfortunately, most photocatalyst materials have band structures far from ideal: some (such as TiO_2_) with band gaps too wide to effectively utilize the solar spectrum, and others (such as transition metal sulfides) with suitable band gaps but improper CB or VB edges for driving CO_2_ reduction or water oxidation. Band structure engineering therefore is frequently sought as a possible strategy to enhance the visible light activity of photocatalysts. It is commonly accomplished via ion doping: cation doping generally modifies the CB, and anion doping modifies the VB. Much knowledge has been accumulated on this approach.

Taking TiO_2_ for example, studies were shown that the cation doping of TiO_2_ by V, Cr, Mn, Fe, and Ni afforded an obvious redshift of its absorption band, and the extent of the shift depended on the amount and the type of the doping.^39^ It is suggested that the doping results in the formation of some localized states below the CB edge of Ti 3d orbitals and consequently obvious band gap narrowing.^116^ Ye and co‐workers reported that the codoping of mesoporous TiO_2_ altered both its CB and VB structure and increased its visible light absorption.^174^ The main CO_2_ reduction products were CO and CH_4_, and their selectivity could be tuned by adjusting the Co doping level. An optimal activity of 90 µmol g^−1^ h^−1^ for CH_4_ and 1.94 mmol g^−1^ h^−1^ for CO was achieved at the Co/Ti molar ratio of 2.5%.

**Figure 11:**
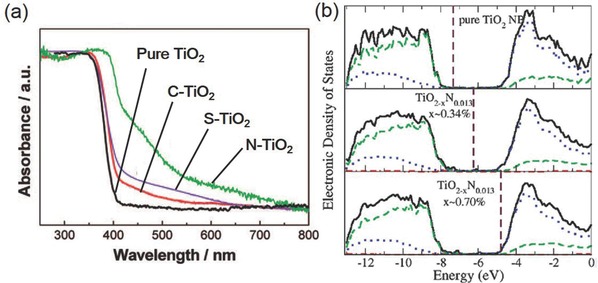
a) Diffuse reflectance spectra of pure TiO_2_, C‐doped TiO_2_, S‐doped TiO_2_, and N‐doped TiO_2_ showing the prominent effect of anion doping. Reproduced with permission.^175^ Copyright 2008, American Chemical Society. b) Calculated density of state (DOS) of pure TiO_2_ and N‐doped TiO_2_ with different concentrations of O vacancies. Reproduced with permission.^176^ Copyright 2009, American Chemical Society.

Compared to cation doping, anion doping is even more desirable. This is because for most oxides (e.g., TiO_2_, ZrO_2_, Nb_2_O_5_, and In_2_O_3_), there is a large room for raising their VB edges without compromising their capability to oxidize water. Partially replacing O in the lattice with other nonmetal elements such as B, C, N, S, and P is proven effective (**Figure** **11**a).^175^ Additional electronic states above the valence band edge introduced by the nonmetal doping were found responsible for the visible‐light response as corroborated by DFT calculations (Figure 11b) and X‐ray photoelectron spectroscopy characterizations.^175^, ^176^ Among various nonmetal doping systems, N‐doping is the most widely studied. Asahi et al. first reported that N‐doping of TiO_2_ could lead to a significant band‐gap narrowing.^177^ The resultant yellowish film exhibited a much improved optical absorption at a wavelength of less than 500 nm. Most remarkably, Cheng and co‐workers demonstrated that red anatase TiO_2_ with a band gap as small as 1.94 V could be prepared by high‐concentration B/N codoping.^178^ Its light absorption edge was accordingly extended up to ≈700 nm covering the full visible light spectrum. The band structure of such a B/N codoped TiO_2_ was close to the ideal state for efficient photocatalysis. Several types of N‐doped TiO_2_ were previously investigated for photocatalytic CO_2_ reduction. N‐doped mesoporous TiO_2_ was developed by Li et al.^179^ With the introduction of 0.2 wt% Pt as the cocatalyst, this photocatalyst enabled a CH_4_ production rate of 2.9 µmol g_cat_^−1^ h^−1^. Grimes and co‐workers demonstrated that N‐doped TiO_2_ nanotube arrays loaded with both Cu and Pt nanoparticles had a hydrocarbon production rate of 111 ppm cm^−2^ h^−1^ or 160 µL g^−1^ h^−1^ under outdoor global AM 1.5 sunlight.^180^

#### 4.2.2 Nanostructure Design

Rapid spatial separation of photogenerated electrons and holes hold the second decisive key to high‐performance photocatalysis. The average distance that photogenerated carriers migrate from the bulk to the surface is known as the diffusion length. If the diffusion length could be significantly shortened to the nanoscale, the probability of charge separation would be dramatically enhanced and that of charge recombination would be suppressed.^181^ As a result, proper nanostructure design of photocatalysts is beneficial to their photocatalytic performance. Nanostructured materials have large specific surface areas and short diffusion length. Their photogenerated carriers more likely reach the surface and participate in the surface electrochemical reaction before the recombination takes place.

**Figure 12:**
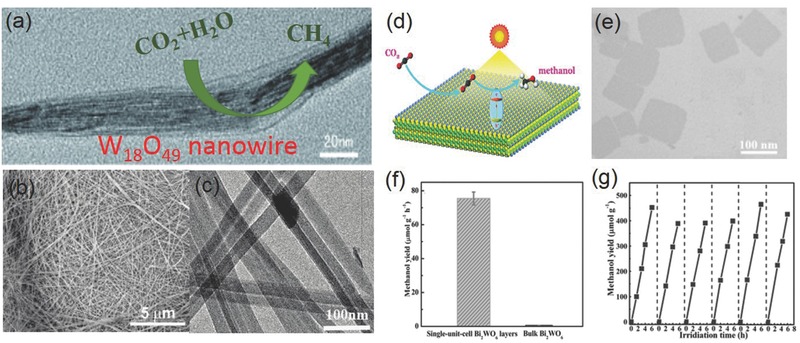
a) TEM image of W_18_O_49_ nanowires for selectively reducing CO_2_ to CH_4_. Reproduced with permission.^182^ b,c) SEM and TEM images of Zn_2_GeO_4_ nanoribbons. Reproduced with permission.^134^ Copyright 2010, American Chemical Society. d) Schematic of the photocatalytic CO_2_ reduction to methanol on the single‐unit‐cell Bi_2_WO_6_ layers; e) TEM image of Bi_2_WO_6_ layers; f) methanol formation rate on Bi_2_WO_6_ layers and bulk Bi_2_WO_6_, g) stability of methanol formation on Bi_2_WO_6_ layers. Reproduced with permission.^183^

A myriad of nanostructured materials at different dimensions are developed for photocatalytic CO_2_ reduction. 1D nanostructures in the form of nanorods, nanowires, nanotubes, and nanobelts have attracted great interest. These materials are usually of single‐crystalline phase that can enable the rapid transport of photogenerated charges. For example, Ye and co‐workers prepared ultrathin W_18_O_49_ nanowires with diameter below 1 nm (**Figure** **12**a).^182^ They showed strong light absorption from the visible to the near‐infrared region, and was able to reduce CO_2_ to CH_4_ in the absence of any cocatalyst at an impressive formation rate of 666 ppm g^−1^ h^−1^. Zou and co‐workers prepared single‐crystalline Zn_2_GeO_4_ nanobelts that was featured with thickness as small as ≈7 nm and aspect ratio up to 10 000 (Figure 12b,c).^134^ Their high crystallinity and ultralong/ultrathin geometry configuration facilitated the migration and separation of photogenerated carriers and consequently resulted in an improved photocatalytic activity for reducing CO_2_ to CH_4_.

In recent years, the great potentials of 2D nanostructures such as nanosheets or nanoflakes start to be gradually unveiled. These 2D materials often have high specific surface areas that can provide abundant active sites for the CO_2_ adsorption and photocatalytic reaction. Xie and co‐workers developed a series of 2D atomic thick semiconductor materials for photocatalytic CO_2_ reduction. They demonstrated that Bi_2_WO_6_ atomic layers could be prepared through a lamellar Bi‐oleate intermediate (Figure 12d,e).^183^ The reduced thickness afforded dramatically improved carrier separation efficiency, as evidenced by the increased carrier lifetime from 14.7 to 83.2 ns based on time‐resolved fluorescence spectroscopy measurements. As a result, the product exhibited a high methanol formation rate of 75 µmol g^–1^ h^–1^—125 times higher than that of bulk Bi_2_WO_6_ and satisfactory stability over 2 d (Figure 12f,g). In their latest report, the same research group followed a similar approach and measured an even better photocatalytic performance for *o*‐BiVO_4_ atomic layers, reaching a remarkable methanol formation rate up to 398.3 µmol g^–1^ h^–1^ and an AQE of 5.96% at 350 nm.^131^

In addition to above 1D and 2D materials, more complex and hierarchical structures have also been developed and demonstrated for photocatalytic CO_2_ reduction. Zou and co‐workers prepared unique hollow spheres composed of single‐layered titanium oxide nanosheets and graphene via layer‐by‐layer assembly.^184^ They displayed enhanced photocatalytic activity, with a CO production rate of 8.91 µmol g^−1^ h^−1^ and a CH_4_ production rate of 1.14 µmol g^−1^ h^−1^, markedly improved over individual titanium oxide nanosheets or P25 TiO_2_. This enhancement was interpreted as the consequence of the ultrathin nature of titanium oxide nanosheets and their intimate contact with graphene, facilitating the rapid charge separation. Their hollow structure was also believed to induce the multiscattering of the incident light and benefit the light adsorption. Yu and co‐workers prepared hierarchical amine‐functionalized titanate yolk@shell microspheres via one‐pot hydrothermal method.^185^ The final product had multilevel porous structures composed of basic building blocks including aggregated nanoparticles as the yolk and self‐assembled 2D nanosheets as the shell. Thanks to the hierarchical structure and amine functionality, the yolk@shell microspheres showed enhanced visible light absorption and CO_2_ adsorption properties, and could selectively reduce CO_2_ to methanol at an impressive rate of ≈2 µmol g^−1^ h^−1^.

#### 4.2.3 Heterostructure Design

**Figure 13:**
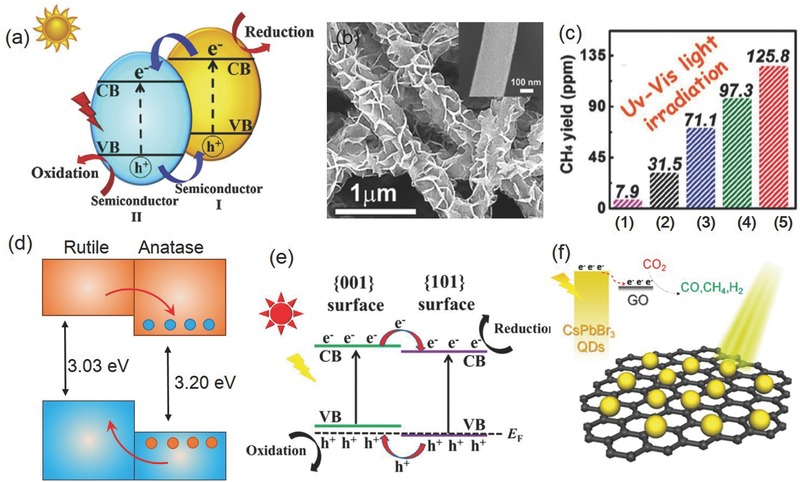
a) Schematic of the conventional type‐II heterojunction photocatalyst. Reproduced with permission.^40^ Copyright 2017, Elsevier. b) SEM image of ZnIn_2_S_4_/TiO_2_; c) comparison of CH_4_ yield from photocatalytic CO_2_ reduction on 1) ZnIn_2_S_4_, 2) TiO_2_, 3) ZnIn_2_S_4_/TiO_2_, 4) Au/ZnIn_2_S_4_/TiO_2_, and 5) Ag/ZnIn_2_S_4_/TiO_2_ after UV–vis irradiation for 4 h. Reproduced with permission.^188^ d) Proposed VB and CB alignment for the anatase/rutile interface. Reproduced with permission.^197^ Copyright 2013, Nature Publishing Group. e) Schematic {001}/{101} surface heterojunction. Reproduced with permission.^199^ Copyright 2014, American Chemical Society. f) Schematic of CO_2_ photoreduction over the CsPbBr_3_ QD/GO photocatalyst. Reproduced with permission.^202^ Copyright 2017, American Chemical Society.

Another strategy to facilitate the spatial separation of photogenerated electrons and holes is via the heterostructure design. When two semiconductor materials are coupled together, they may form three possible types of heterostructures namely straddling gap (type I), staggered gap (type II), and broken gap (type III), depending on their relative band positions.^186^ Among them, the type II of heterosturcture is the most desirable since it promotes the spatial charge separation by transferring electrons to one material with the lower CB and holes to another material with the higher VB, as is shown in **Figure** **13**a.^187^ Coupling semiconductors with staggered gaps is an approach frequently adopted in photocatalysis. TiO_2_ is a common component of many heterostructures. Dong and co‐workers described a hierarchical assembly of ultrathin ZnIn_2_S_4_ nanosheets on TiO_2_ electrospun nanofibers (Figure 13b–d).^188^ These two components had low lattice mismatch and suitable band alignment to form type II heterostructure. The fast separation of photogenerated carriers was attested by the reduced decay lifetime and quenched ZnIn_2_S_4_ photoluminescence signal. When further functionalized with plasmonic Au or Ag nanoparticles, the optimal photocatalyst exhibited ≈16‐fold improvement for the photocatalytic reduction of CO_2_ to CH_4_ compared to pure ZnIn_2_S_4_. N‐type TiO_2_ can also be coupled with p‐type semiconductors such as CuO or Cu_2_O to form type II p−n hereojunctions.^189^, ^190^, ^191^, ^192^ Schaak and co‐workers synthesized a CuO‐TiO_2−_ *_x_*N*_x_* p–n junction though the reactive template method.^193^ It photocatalytically reduced CO_2_ to methane under simulated solar irradiation at a rate of 41.3 ppm g^−1^ h^−1^—much enhanced over pure CuO or TiO_2−_ *_x_*N*_x_*. Apart from TiO_2_, silver halides are also frequently investigated for forming heterostructures.^194^ Chai and co‐workers developed AgX/pCN (X = Cl and Br) heterojunction photocatalysts by depositing AgX (X = Cl and Br) on protonated graphitic carbon nitride.^195^ The optimal photocatalyst achieved a total CH_4_ evolution rate of 10.92 µmol g^−1^ h^−1^, which were 34.1 and 4.2 times higher than individual AgBr and pCN respectively due to the improved separation efficiency of photogenerated electron/hole pairs at the heterojunction interface.

In addition to heterostructures formed from materials with distinctive compositions, this concept may also be extended to some single‐component systems (sometimes termed homojunction), where different phases or surface facets coexist, and have varying energetics to allow for the spatial separation of photogenerated carriers.^196^ A good example is the anatase and rutile polymorphs of TiO_2_. When brought together, they form a type II, staggered band alignment with ≈0.4 eV difference in band position.^197^ Such a difference is significant enough to promote the migration of electrons from rutile to anatase, and holes from anatase to rutile, rendering the mixed‐phase photocatalyst generally superior to individual polymorphs. Another example is the homojunction formed by α/β phase Ga_2_O_3_ showing over three folds higher activity for photocatalytic water splitting than individual α‐ or β‐Ga_2_O_3_.^198^ The influence of different surface facets is also documented in literature. Jaroniec and co‐workers showed that the {101} and {001} facets of anatase TiO_2_ exhibited slightly different band edge positions based on the DFT calculations (Figure 13e).^199^ The coexistence of these two facets in single TiO_2_ particle would create a surface heterojunction, which facilitated the migration of the photogenerated electrons and holes to the {101} and {001} facets, respectively (Figure 13f). The ratio of different crystal facets substantially influenced the photocatalytic activity of TiO_2_, and the optimal {101}/{001} ratio was experimentally identified to be 45:55 for the photocatalytic conversion of CO_2_ to CH_4_.

Furthermore, even though carbon nanomaterials (e.g., graphene or carbon nanotubes) are typically not light responsive, they are often employed to form hybrid materials with light‐absorbing semiconductor photocatalysts, which can also be regarded as a special type of heterostructures. These carbon nanomaterials have large surface areas for supporting photocatalysts, may enable fast extraction of photogenerated carriers from the semiconductor, and hence improved photocatalytic activity. There are many successful demonstrations along this line.^200^, ^201^ For example, in a most recent report, Kuang and co‐workers showed that supporting CsPbBr_3_ quantum dots on graphene oxide considerably improved their photocatalytic activity, reaching an average CO formation rate of 4.9 µmol g^−1^ h^−1^ and CH_4_ formation rate of 2.1 µmol g^−1^ h^−1^ under AM 1.5G simulated illumination.^202^

#### 4.2.4 Defect Engineering

**Figure 14:**
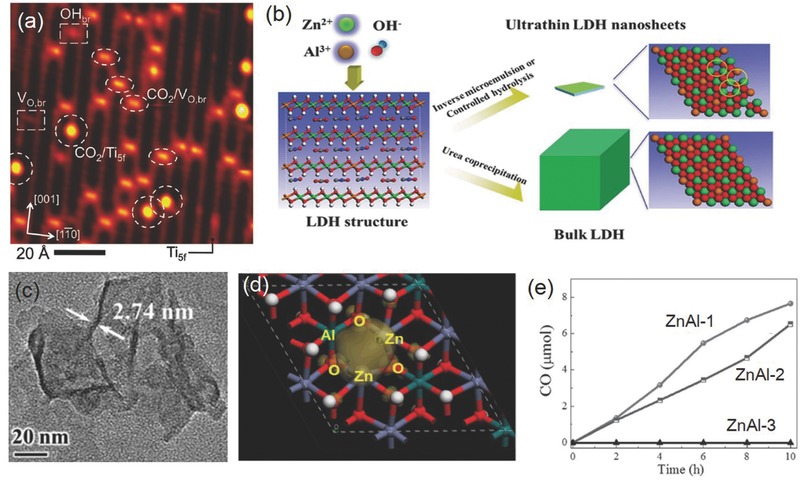
a) Scanning tunneling microscope (STM) image of CO_2_ molecules adsorbed on TiO_2_ (110) plane. Reproduced with permission.^206^ Copyright 2011, American Chemical Society. b) Schematic showing the formation of coordinatively unsaturated ZnAl‐LDH nanosheets; c) TEM image of coordinatively unsaturated ZnAl‐LDH nanosheets; d) charge density distribution for the valence band maximum of V_o_‐doped ZnAl‐LDH; e) time‐dependent CO yields on different ZnAl‐LDH samples. Reproduced with permission.^159^

Defects play a crucial role in catalysis. It may change the interaction between the catalyst surface and the target molecule, consequently lower the reaction activation energy or even alter the reaction pathway. Engineering the type and density of defects on catalyst surfaces is an important means to tune their activities. Oxygen vacancies are among the most common defects in oxide or hydroxide surfaces and are suggested to greatly affect the photocatalytic CO_2_ reduction.^203^, ^204^, ^205^ Among many experimental supports was the low‐temperature scanning tunneling microscopy visualizing that CO_2_ molecules were preferably absorbed at the oxygen vacancies (**Figure** **14**a).^206^ Quantum mechanical modeling also indicated that the electron transfer from the CB of stoichiometric anatase TiO_2_ to CO_2_ was not energetically favorable, but defects on anatase TiO_2_ surface could promote the electron transfer to CO_2_.^204^

Many semiconductor photocatalysts containing rich structural defects have been investigated for CO_2_ reduction with improved performances. Wang and co‐workers showed that the CO_2_ photoreduction over defective CeO_2_ selectively yielded CO at a decent formation rate of ≈4 µmol g^–1^ h^–1^, and eliminating these defects by postsynthetic annealing fully deactivated the photocatalyst.^207^ The authors proposed that oxygen vacancies together with local strain promoted the CO_2_ capture and activation on the CeO_2_ surface, therefore lowering its reaction barrier. Self‐doped SrTiO_3−_ *_δ_* powders were prepared by Ye and co‐workers and analyzed to contain Ti^3+^ ions and oxygen vacancies by forming Ruddlesden–Popper crystallographic shears.^205^ These defects not only induced an in‐gap band to enhance the visible light absorption of the photocatalyst but also improved the chemical adsorption of CO_2_ on the surface based on temperature programmed desorption experiments. A better photocatalytic activity for converting CO_2_ to CH_4_ was therefore resulted. Zhang and co‐workers recently reported that abundant oxygen vacancies and coordinatively unsaturated Zn^+^ centers were created when the thickness of ZnAl LDH nanosheets were reduced to two repeat stacking layers (Figure 14b–d).^159^ Thus formed Zn^+^‐V_o_ complexes served as the active sites for the efficient adsorption of CO_2_ and H_2_O molecules, significantly improving the photocatalytic activity for CO_2_ reduction to CO.

#### 4.2.5 Cocatalyst Loading

Even when photogenerated carriers are rapidly separated and migrate to the surface, they may not readily participate in the surface redox reactions since both CO_2_ reaction and water oxidation involve multistep proton‐coupled electron transfer and are notorious for their highly sluggish nature. A common and often necessary strategy to improve the photocatalytic performance is to introduce auxiliary cocatalysts to the surface of the semiconductor photocatalyst.^208^ These cocatalysts markedly change the energetics of the charge transfer process at the surface and increase the catalytic turnover rates, making the production rate of solar fuels a dominant process over the charge recombination or reverse reactions.^209^ In addition, the timely consumption the photogenerated charges on the cocatalyst would also slow down the photocorrosion of semiconductor photocatalysts and improve their stability. As reviewed in Section 3, many different CO_2_RR electrocatalysts have been developed in the last five years. In principle, they are all potential cocatalyst materials for photocatalytic CO_2_ reduction, provided a favorable interface is established between the photocatalyst and cocatalyst to facilitate the charge transfer from the former to the latter.

**Figure 15:**
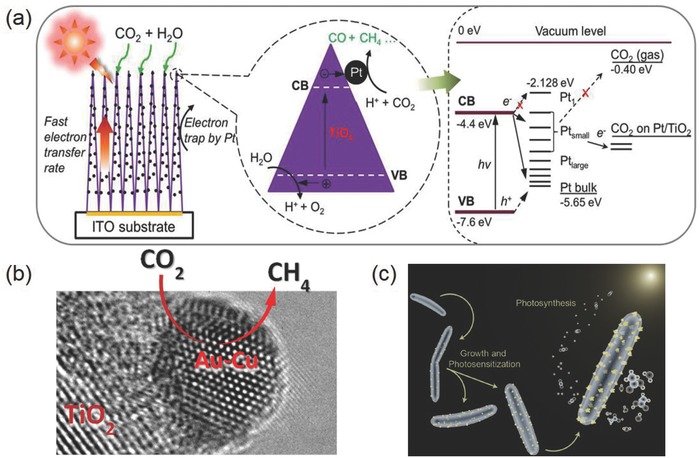
a) Schematic of photocatalytic CO_2_ reduction on nanostructured TiO_2_ films deposited with Pt cocatalyst particles of varying sizes. Different alignments between TiO_2_ band structure and Pt work function is suggested to be responsible for the observed different photocatalytic activities. Reproduced with permission.^211^ Copyright 2012, American Chemical Society. b) High‐resolution TEM image of an Au−Cu nanoparticle deposited on the TiO_2_ surface as the cocatalyst for selectively reducing CO_2_ to CH_4_. Reproduced with permission.^212^ Copyright 2014. American Chemical Society. c) Schematic showing the *M. thermoacetica*–CdS hybrid system for the photosynthetic conversion of CO_2_ to acetic acid. Reproduced with permission.^213^ Copyright 2016, American Association for the Advancement of Science.

Noble metals including Pt, Au, Pd, and Ag represent the most widely used cocatalyst materials for photocatalytic CO_2_ reduction. They are usually deposited onto the photocatalyst surface via either chemical reduction or photochemical reduction of corresponding precursors. These noble metals can often serve as the electron sink to concentrate photogenerated electrons from photocatalysts, and consequently reduce the possibility of electron‐hole recombination.^209^ Within their presence, the selectivity of CO_2_ photoreduction is generally shifted in favor of CH_4_ over CO or other hydrocarbon products. Using P25 TiO_2_ as the model photocatalyst, Wang and co‐workers demonstrated that the ability of noble metal cocatalysts to promote the CH_4_ formation rate increased in the order Ag < Rh < Au < Pd < Pt.^210^ The most effective cocatalyst was identified to be Pt, presumably due to its efficient extraction of photogenerated electrons from TiO_2_. It was also recognized that the size of cocatalyst particles affected the photocatalytic activity and selectivity. CH_4_ formation was more favored on smaller Pt nanoparticles. TiO_2_ loaded with ≈3 nm (mean size) Pt nanoparticles was measured to produce CH_4_ about four times faster than TiO_2_ loaded with ≈5 nm Pt nanoparticles. Additional introduction of MgO in the photocatalyst further doubled the CH_4_ formation rate and boosted its selectivity to >99%. Biswas and co‐workers also studied the size effect of Pt cocatalyst on the photocatalytic performance of TiO_2_.^211^ The optimal particle size was measured to be ≈1 nm. With such a cocatalyst particle size, a peak CH_4_ formation rate of 1361 µmol g^–1^ h^–1^ was observed, and the corresponding quantum yield was calculated to be 2.41% (**Figure** **15**a). By contrast, larger or smaller cocatalyst nanoparticles led to markedly reduced photocatalytic activity. The difference was rationalized based on the relative energy band alignment of TiO_2_ and Pt as its size varied: too small Pt particle size would lift its energy level above the bottom of the TiO_2_ CB due to the quantum confinement, retarding the electron transfer from photocatalyst to Pt cocatalyst, whilst for bigger Pt nanoparticles, their properties approached bulk Pt and could act as recombination centers by capturing both photogenerated electrons and holes.

Aside from monometallic cocatalysts, bimetallic cocatalysts are also considered for photocatalytic CO_2_ reduction. As previously mentioned, alloying of different metals offers the probability to tailor their electrocatalytic activity and selectivity. Garcia and co‐workers showed that Au–Cu nanoalloy cocatalyst greatly promoted the photocatalytic activity of commercial P25 TiO_2_ (Figure 15b).^212^ The optimal Au/Cu ratio was determined to be 1: 2. Under this condition, the photocatalyst exhibited an excellent CH_4_ formation rates up to 2.2 mmol g^−1^ h^−1^ and minimal concomitant H_2_ generation.

Very recently, it was showed bacterial or enzymatic materials might also be used as the cocatalyst to promote the performance of photocatalysts. The proof of concept was first demonstrated by Yang and co‐workers by integrating the non‐photosynthetic CO_2_‐reducing bacterium *M. thermoacetica* with CdS nanoparticles (Figure 15c).^213^ The hybrid system selectively (≈90%) photoreduced CO_2_ to acetic acid. The optimal production rate was reported to be ≈520 × 10^−6^ m h^−1^ with a quantum yield of ≈52% under 485 nm light illumination. This study represented an important leap in the pursuit of efficient artificial photosynthesis and uncovered the great potential of inorganic‐biological hybrid systems.

#### 4.2.6 Z‐Scheme System Construction

**Figure 16:**
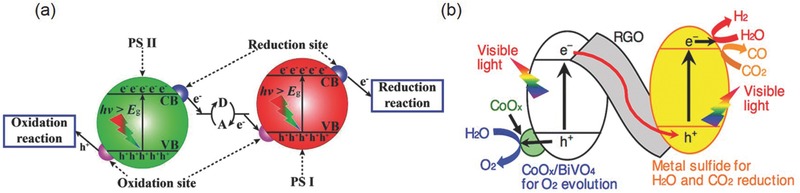
a) Schematic of Z‐scheme photocatalytic mechanism. Reproduced with permission.^216^ b) Schematic of the Z‐scheme system for water splitting and CO_2_ reduction by coupling Pt‐loaded metal sulfide and CoO*_x_*/BiVO_4_ using RGO as the solid state electron mediator. Reproduced with permission.^220^ Copyright 2014, American Chemical Society.

Suitable band structure, efficient charge separation, and rapid surface charge transfer are key characteristics of successful photocatalysts. Yet it is highly challenging (if not impossible) for a single‐component photocatalyst to simultaneously meet all these criteria. In nature, photosynthesis takes place in two individual but well concerted steps in photosystems I and II that harvest 700 and 680 nm photons, respectively.^214^, ^215^ The sites for oxygen evolution and CO_2_ fixation are also spatially separated. Such a natural two‐step process inspires the design of Z‐scheme systems for artificial photosynthesis.^216^ Instead of using a single‐component photocatalyst to fulfill all the functions, one may combine two different light‐absorbing semiconductor materials through a redox mediator, which electrically connects the two parts by mediating the electron transfer from the CB of one semiconductor with relatively lower energy to the VB of another semiconductor (**Figure** **16**a). In this configuration, the cathodic and anodic half reactions can be decoupled, and the selection criteria for photocatalyst materials can be considerably relaxed. Z‐scheme can more efficiently utilize visible light, capitalize the strongly reducing electrons from one catalyst and the strongly oxidizing holes from another, and achieve overall photocatalytic activities that are not attainable with single‐component systems. Many successful Z‐scheme designs are now available. Arai and co‐workers constructed a Z‐scheme system by combining InP/Ru complex polymer (for CO_2_ reduction to formate) with TiO_2_ (for water oxidation).^217^ The selectivity for formate reached >70% and the overall conversion efficiency from solar energy to chemical energy was 0.03–0.04%, which approached that of natural photosynthesis in plants. The conversion efficiency was further increased to 0.14% in a follow‐up work by the same research group when TiO_2_ in the Z‐scheme was replaced by SrTiO_3_ since its higher conduction band facilitated the electron transfer to InP.^218^ Sulfide‐based photocatalysts are generally susceptible to photocorrosion. Z‐scheme is an effective strategy to alleviate their photocorrosion by rapidly filling the photogenerated holes in the VB of sulfide photocatalysts. Kudo and co‐workers studied the combination of several different p‐type sulfide photocatalysts (such as ZnS, AgGaS_2_, AgInS_2_, CuGaS_2_, CuInS_2_, and many others) and n‐type oxide photocatalysts (such as TiO_2_ and BiVO_4_) in the Z‐scheme configuration for photocatalytic water splitting or CO_2_ reduction, using RGO as the solid state electron mediator (Figure 16b).^219^, ^220^ In particular, the combination of CuGaS_2_ and BiVO_4_ was demonstrated to reduce CO_2_ to CO with a good short‐term stability even though it was accompanied by a significant cogeneration of H_2_. The low CO selectivity could probably be improved by introducing proper cocatalysts. Z‐scheme may also be formed between a molecular photosensitizer and a semiconductor photocatalyst.^221^ Ishitani and co‐workers constructed a hybrid photocatalyst by combining Ru(II) binuclear complex and Ag‐loaded TaON together with organic hole scavenger.^222^ HCOOH was detected as the main reduction product. Its maximum TON for HCOOH was measured to be 750 under visible‐light irradiation for 24 h, and its optimal external quantum efficiency was 0.48% at 400 nm.

## 5 Conclusion and Perspectives

This review summarizes recent advances in CO_2_ reduction to useful chemical fuels via the electrochemical or photochemical approach. Even though some significant advances have been achieved in the past decades for both electrocatalytic and photocatalytic CO_2_ reduction, their reaction activity and selectivity are still rather low. At this moment, there is no detailed technoeconomical analysis available to estimate the target production cost of chemical fuels from CO_2_ reduction in order to compete with other fuel production technologies. For water splitting, US Department of Energy has specified the solar‐to‐hydrogen commercialization target is $2.00–$4.00 per kg of H_2_ and the minimum solar energy conversion efficiency should be 5% and preferably >10% to compete with gasoline.^223^ We therefore envisage that a comparable solar‐to‐fuel efficiency is necessary for photocatalytic or PV+electrocatalytic CO_2_ reduction to become a commercial reality. Even though the solar to fuel (STF) efficiency of most current CO_2_ reduction systems is still well below the minimum target, two recent breakthroughs are worth highlighting here. By integrating a photovoltaic cell with biocompatible Earth‐abundant inorganic catalysts and bacterium *Ralstonia eutropha*, Nocera and co‐workers achieved an artificial photosynthetic process for carbon fixation into biomass and liquid fuels at an efficiency of ≈10%.^224^ Gratzel and co‐workers demonstrated atomic layer deposition of SnO_2_ on CuO nanowires as a bifunctional electrocatalyst for both CO_2_RR and OER, and when coupled with a GaInP/GaInAs/Ge photovoltaic cell, enabled an solar‐to‐CO conversion efficiency of 13.4%.^225^ The efficiencies reported in the above two studies already exceed that of natural photosynthetic systems and are suggestive of the great potential of the CO_2_ reduction technology.

To further enhance the CO_2_ reduction performance, improvements can be possibly made from the following two directions. Seeking new material compositions and structures would continue to be at the heart of electrocatalytic and photocatalytic CO_2_ reduction research. Priority should be given to exploration guided by theoretical computation. Ample examples have been shown that theoretical computation is a powerful tool in predicting new catalysts by comparing the energy barriers and overpotentials of reaction intermediates on certain crystal surfaces of each catalyst. In the future, the subjects of computation may be extended from currently predominant metallic catalysts (due to their well‐defined surface geometry) to more complicated systems including pure compounds (oxides, sulfides, and so on) or even hybrids. For photocatalytic CO_2_ reduction, the exploration of new materials and structures can also be greatly accelerated by borrowing knowledge from photocatalytic water splitting. Photocatalytic CO_2_ reduction and photocatalytic water splitting only differs in their surface reaction step. If strategies (such as incorporation of proper cocatalysts) can be undertaken to significantly shift the cathodic reaction selectivity away from HER to CO_2_RR, essentially all existing photocatalysts for water splitting can be transformed to those for CO_2_ reduction.

Increasing understanding of the reaction pathway and mechanism would help us toward the design of better CO_2_ reduction catalysts. A clear picture of how CO_2_ is adsorbed, transformed and desorbed is still missing at this moment. Of particular interest is to understand the rate determining step in CO_2_ reduction and the surface binding of its reaction intermediates so that we can accordingly strengthen or weaken the intermediate binding and expedite the overall reaction rate. Many advanced electronic and spectroscopic tools such as aberration‐corrected transmission electron microscopy (TEM), X‐ray absorption spectroscopy, electron spin resonance, and time‐resolved fluorescence spectroscopy are now available to us with unprecedented capabilities. They may offer detailed information about the catalyst structure at different levels and insights about the catalytic process. We will also see more and more in situ characterization techniques such as in situ Raman and attenuated total reflection infrared spectroscopy brought in to shed new light on the binding configuration and environment of reaction intermediates.

CO_2_ reduction is of both fundamental and practical significance. In spite of serious challenges, there is no reason to doubt its great potential and impact. More efforts are called for in the fundamental understanding, materials development, and catalyst engineering of CO_2_ reduction to make it a viable technology for a carbon neutral future.

## Conflict of Interest

The authors declare no conflict of interest.
